# Metagenomic and metabolomic analysis showing the adverse risk–benefit trade-off of the ketogenic diet

**DOI:** 10.1186/s12944-024-02198-7

**Published:** 2024-06-29

**Authors:** Hongyan Qiu, Chengxia Kan, Fang Han, Youhong Luo, Na Qu, Kexin Zhang, Yanhui Ma, Ningning Hou, Di Wu, Xiaodong Sun, Junfeng Shi

**Affiliations:** 1 Department of Endocrinology and Metabolism, Affiliated Hospital of Shandong Second Medical University, Weifang, 261031 China; 2 Clinical Research Center, Affiliated Hospital of Shandong Second Medical University, Weifang, 261031 China; 3 Department of Pathology, Affiliated Hospital of Shandong Second Medical University, Weifang, 261031 China

**Keywords:** Obesity, Ketogenic diet, High-fat diet, Gut microbiota, Metagenome, Metabolome

## Abstract

**Background:**

Ketogenic diets are increasingly popular for addressing obesity, but their impacts on the gut microbiota and metabolome remain unclear. This paper aimed to investigate how a ketogenic diet affects intestinal microorganisms and metabolites in obesity.

**Methods:**

Male mice were provided with one of the following dietary regimens: normal chow, high-fat diet, ketogenic diet, or high-fat diet converted to ketogenic diet. Body weight and fat mass were measured weekly using high-precision electronic balances and minispec body composition analyzers. Metagenomics and non-targeted metabolomics data were used to analyze differences in intestinal contents.

**Results:**

Obese mice on the ketogenic diet exhibited notable improvements in weight and body fat. However, these were accompanied by a significant decrease in intestinal microbial diversity, as well as an increase in Firmicutes abundance and a 247% increase in the Firmicutes/Bacteroidetes ratio. The ketogenic diet also altered multiple metabolic pathways in the gut, including glucose, lipid, energy, carbohydrate, amino acid, ketone body, butanoate, and methane pathways, as well as bacterial secretion and colonization pathways. These changes were associated with increased intestinal inflammation and dysbiosis in obese mice. Furthermore, the ketogenic diet enhanced the secretion of bile and the synthesis of aminoglycoside antibiotics in obese mice, which may impair the gut microbiota and be associated with intestinal inflammation and immunity.

**Conclusions:**

The study suggest that the ketogenic diet had an unfavorable risk–benefit trade-off and may compromise metabolic homeostasis in obese mice.

**Supplementary Information:**

The online version contains supplementary material available at 10.1186/s12944-024-02198-7.

## Introduction

Obesity and overweight are chronic and heterogeneous metabolic disorders that are associated with a wide range of medical conditions, including diabetes mellitus, insulin resistance and tumors [1, 2]. More than 2 billion people are overweight or obese, representing approximately 30% of the world’s population [3]. Given the rapid increase in the prevalence of obesity and its devastating impact on health, addressing this issue with existing treatments as early as possible is of paramount importance.

Because obesity is a consequence of a mismatch in energy assimilation and expenditure, many factors need to be considered in its treatment. Currently, there are limited approved anti-obesity drugs, which include liraglutide injection, phentermine, bupropion sustained release, lorcaserin, topiramate/phentermine extended release, and orlistat [4]. Although these drugs generally reduce appetite or increase satiety through the central nervous system, further consideration of their safety and efficacy profiles is required [4]. Additionally, such drugs are associated with certain adverse events, including gastrointestinal problems, headache, irritability, difficulty sleeping, blurred vision, and hypertension [5–8]. Therefore, while healthy and effective approaches to improve the imbalance in energy absorption and expenditure in obesity are urgently needed, dietary interventions have garnered greater attention.

The various diets that have been proposed for weight loss include high/low carbohydrate diets, very-low-calorie diets, meal replacers, low protein diets, and low glycemic index diets [9, 10]. Notably, the assimilation velocities of different macronutrients depend on their complexity and hormonal kinetics. A low-carbohydrate, high-fat ketogenic diet utilizes fat and 10% protein as the primary energy sources [11]. Although the long-term metabolic effects and underlying mechanisms of ketogenic diets remain unclear, they have demonstrated effectiveness in epilepsy patients, with recent reports also suggesting their efficacy for treatment of obesity [12–15].

The gut microbiota is essential for host metabolism and is highly sensitive to dietary intake [16, 17]. There is clear evidence that 10% protein intake is sufficient for normal growth of mice [18]; thus, the effects of a ketogenic diet on the gut primarily stem from fat and carbohydrates, rather than protein.

Olson et al. and Mardinoglu et al. reported that a ketogenic diet alters the structure and function of the gut microbiota [19, 20]. Alterations in gut microbiota composition and function are recognized hallmarks of metabolic dysfunction [21]. Accumulated evidence suggests that ketogenic diets impact metabolism and metabolic disorders through the gut microbiota, which serves as a regulatory factor of various aspects of host metabolism [22]. The gut microbiota produces a highly diverse array of metabolites and plays critical roles in host processes, including maintenance of energy homeostasis and lipid metabolism. Multiple studies indicate that the gut microbiota significantly contributes to obesity, insulin resistance, and functional disorders [23, 24]. Nevertheless, the subtle mechanistic relationships between ketogenic diets and the abundances of gut microbes and their metabolites in obesity also require further elucidation. Therefore, a comprehensive investigation of the interplay between a ketogenic diet and the gut microbiota in the context of obesity was conducted by analyzing the metagenomes and metabolomes of mice fed with a normal diet, high-fat diet, ketogenic diet, or high-fat diet converted to a ketogenic diet. The primary objective was to elucidate the chemical communication between the ketogenic diet and the gut microbiota to establish a more comprehensive mechanistic understanding of the impact of this diet on host physiology during obesity. These results offer novel insights into the relevance between gut microbiota metabolites and metabolic dysfunction in obesity.

## Methods

### Animals

The animal experiment was approved by the Animal Ethics Committee of Weifang Medical University (2021SDL131) and followed the relevant laws. A cohort of 32 4-week-old male C57BL/6J mice (Pengyue, Jinan, China) was used in this study (single gender was selected to control potential gender-based variability in response to the dietary interventions). The mice were randomly divided into four groups: the normal diet (ND) group was fed a diet comprising 65% carbohydrates (corn starch and sucrose), 20% protein (casein), and 15% fat (soybean oil, lard) (*n* = 8); the high-fat diet (HFD) group was fed 60% fat (soybean oil, lard), 20% carbohydrates (corn starch and sucrose), and 20% protein (casein) (*n* = 8); the ketogenic diet (KD) group was fed 90% fat (soybean oil, lard) and 10% protein (casein) (*n* = 8); and the high-fat to ketogenic conversion (HK) group was transitioned from the high-fat diet to the ketogenic diet described above (*n* = 8). The cellulose content of each diet was the same: 50 g%. During the experiment, food intake was recorded weekly.

ND, HFD, and KD groups were maintained for 20 weeks under the corresponding feeding conditions. The HK group was fed with the high-fat diet for 12 weeks and then converted to the ketogenic diet for another 8 weeks. All mice were housed in a standard environment. Body weight and fat were measured using a Bruker Minispec LF50 device (Bruker, Karlsruhe, Germany). The conditions of the Minispec instrument were below: 90º pulse length, 14.88 µs; 180ºpulse length, 29.64 µs; pulse attenuation, 6 dB; detection angel, 54º; gain, 57 dB; magnetic field steps, 261; rec. dead time, 0.03392 ms; recycle delay, 2 s; field homog. Limit, 0.1 ms; desired magnet temp, 37℃, Nuclear Magnetic Resonance Spectroscopy frequency, 7.5 MHz. Mice were euthanized with 200 mg/kg sodium pentobarbital to collect the contents of the colon and other tissues.

### Metagenomic sequencing and analysis

DNA was isolated from samples (*n* = 3), then DNA libraries were constructed, and the resulting DNA was fragmented. After the library passed quality testing, a NovaSeq™ 6000 (Illumina, San Diego, CA, USA) was used to perform 150-bp paired-end sequencing. Trimmomatic (version 0.40) was used to process raw sequencing data and remove adapters. Low-quality and host sequences were excluded by BMTagger. Metrics of α-diversity, including the observed species and Chao1 indices, were calculated using QIIME v.1.91. The linear discriminant analysis effect size (LEfSe) method, a statistical tool that detects significant differences in features (e.g. genes, organisms, clades, operational taxonomic units, functions) between biological conditions, was performed to identify and visualize differences in microbial communities. Cladograms were visualized using R language. The enrichment analysis was performed by using ClusterProfiler. Sequence taxonomy classification of each group was performed by Kraken2.

### Non-targeted metabolome analysis

#### Metabolite extraction

Intestinal contents from each group (*n* = 6) were extracted using 50% pre-cooled methanol (1:6, v/v) [25], and then analyzed by Ultra-performance liquid chromatography-tandem mass spectrometry (UPLC-MS/MS). Quality control (QC) samples comprised a combination of 10 µL of each extracted sample.

#### UPLC-MS/MS analysis

UPLC coupled with a Triple-TOF 5600 Plus system (SCIEX, Warrington, UK) was used to analyze samples. An C18 column (10 cm × 2.1 mm, 1.8 μm; Waters, UK) was used for compound separation. The mass spectrometer settings were as follows: Spray voltage: positive, 5000 V; negative, -4500 V; information-dependent acquisition of data; mass range, 60–1200 Da; dynamic exclusion, 4 s; and mass accuracy frequency.

#### Metabolomics data processing

XCMS, CAMERA, and metaX were used to process raw data files. Ion peaks were matched to public reference databases and in-house databases. MetaX was used for peak intensity preprocessing. Principal Component Analysis (PCA) was conducted to detect batch effects and abnormal values. Probabilistic quotient normalization algorithm was applied to normalize data across all samples. QC samples were employed for batch correction using the C-robust spline algorithm. MetaX software was used to supervise partial least squares-discriminant analysis (PLS-DA) of the variables to identify more specific statistics of between-group difference. The threshold of variable importance in projection (VIP) was 1.0.

### Statistical analysis

Data with normal distribution are presented as means ± standard error of mean. Student’s t-tests and One-way analysis of variance were used for comparisons. Microbial α-diversity was analyzed using Wilcoxon rank sum tests. Graphs were generated using R package and Graphpad Prism (San Diego, CA, USA).

## Results

### Ketogenic diet ameliorates high-fat diet-induced obesity

**Fig. 1:**
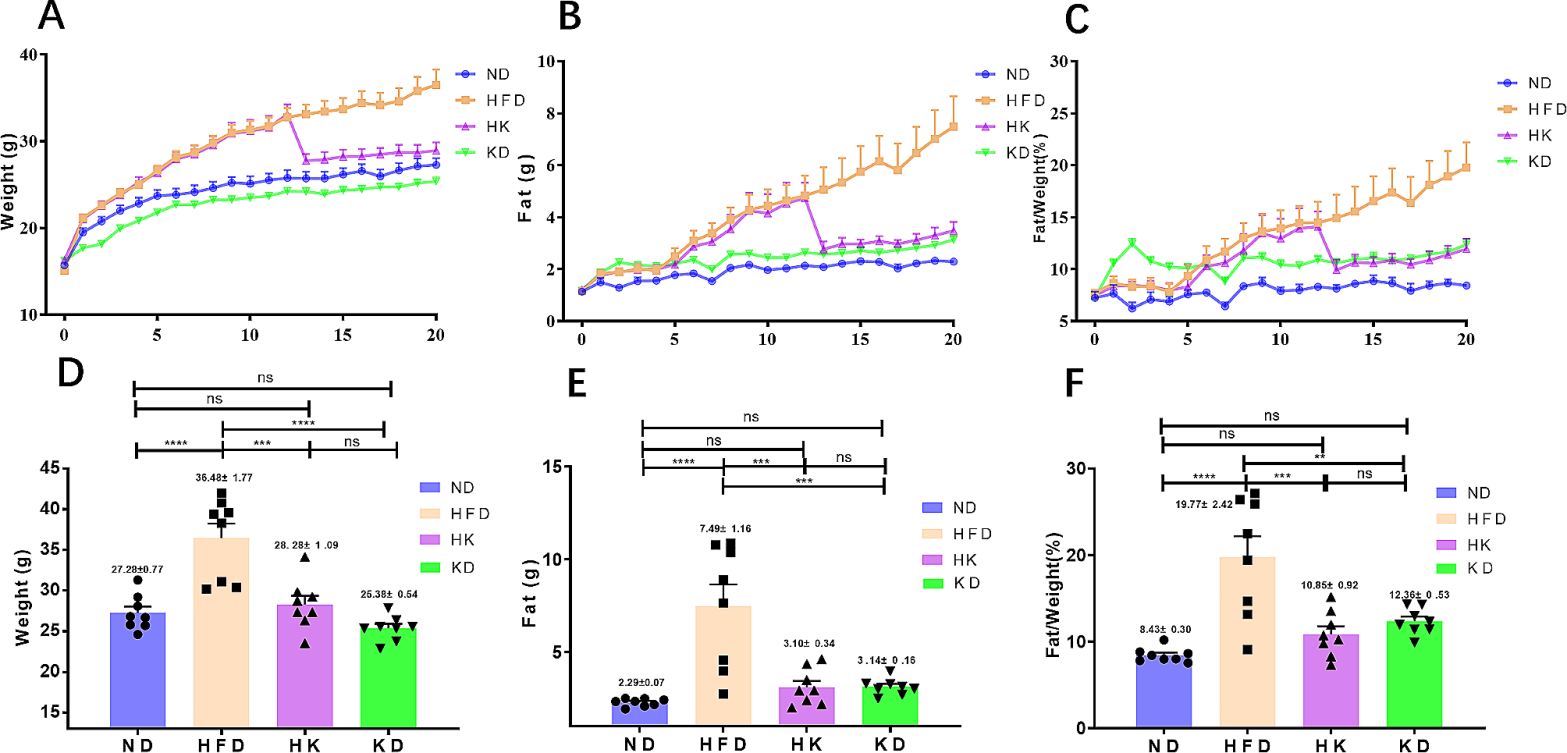
Changes in mice’s body weight, fat mass, and fat/weight% under different feeding conditions. A-C depicts the time-dependent changes in body weight, fat mass, and fat/weight% of mice over 20 weeks. D-F illustrates the body weight, fat mass, and fat/weight% of mice at week 20. **P < 0.01; ***P < 0.001; ****P < 0.0001; ns, not statistically significant; ND, normal diet; HFD, high-fat diet; KD, ketogenic diet; HK, HFD converted to a ketogenic diet; *n* = 8. One-way ANOVA was used to decide significant differences between groups, data were presented as mean ± SEM

After 5 weeks of high-fat diet feeding, mice in HFD and HK groups showed significant increases in body weight, fat mass, and fat/weight% compared to the ND and KD groups (*P* < 0.05; Fig. 1A–C). Notably, switching the HFD group to a ketogenic diet at week 12 led to significant decreases in body weight, fat mass, and fat/weight% (*P <* 0.05). Furthermore, the disparities in these metrics between the HK and ND groups were not statistically significant after one week of this conversion (*P* > 0.05). By the end of the first week, the KD group exhibited a significant reduction in body weight. Despite this weight loss, the fat mass and fat-to-weight ratio in the KD group remained higher than those in the ND group. By the 20th week, the HFD group showed substantial increases in body weight, fat mass, and fat percentage compared to the ND, KD, and HK groups (*P <* 0.05). Conversely, no notable variances these three parameters were observed among the ND, KD, and HK groups (*P >* 0.05; Fig. 1D–F). Supplementary Table 1 provides a more precise visualization of the data shown in Fig. 1, including the assessments of body weight, fat mass, and fat/weight%. No differences were found in food intake (kilocalories) of these four groups (Supplementary Figure S1).

### Intestinal microbial communities

**Fig. 2:**
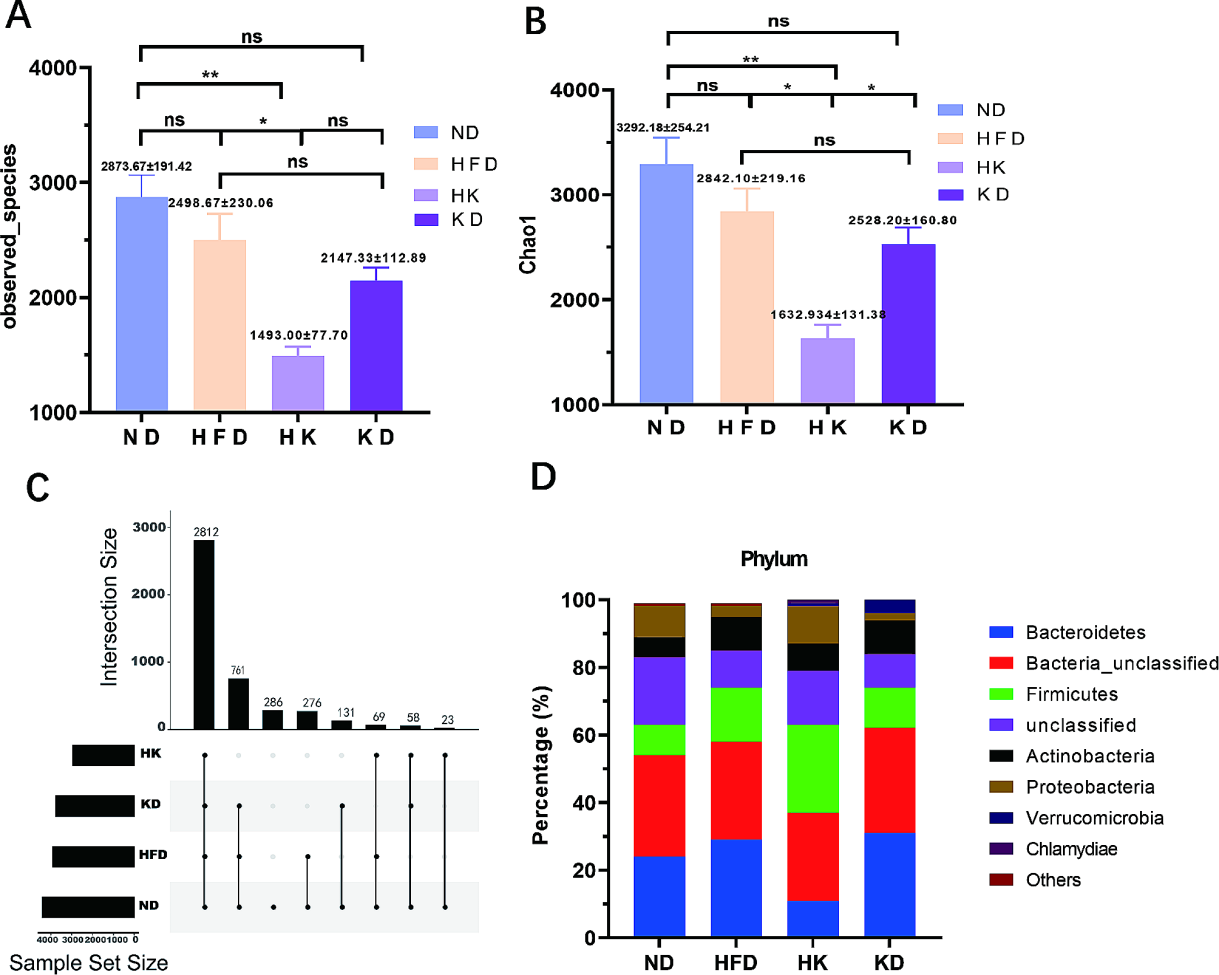
Alpha Diversity, species and abundance of microorganisms. A, observed species at the community level; B, Chao 1 index at the community level; C, the number of intestinal species; D, the mean abundance of gut bacteria at the phylum level. *P < 0.05; **P < 0.01; ns, not statistically significant; *n* = 3. One-way ANOVA was used to decide significant differences between groups, data were presented as mean ± SEM

The diversity of gut microbiota was evaluated by calculate the observed species index and the Chao1 index (Fig. 2A, B). The observed species index counts the number of distinct species identified in a sample. The Chao1 index is a measure of species richness that estimates the total number of species in a community, accounting for both observed species and the number of rare species (singletons and doubletons). Compared with the ND group (observed species, 2873.67 ± 191.42; Chao1, 3292.18 ± 254.21), there tended to be decreases in these indices in the HFD (2498.67 ± 230.06; 2842.10 ± 219.16; *P* > 0.05) and KD (2147.33 ± 112.89; 2528.20 ± 160.80, *P* > 0.05) groups. However, the observed species and Chao1 indices were significantly reduced in HK group (1493.00 ± 77.70; 1632.934 ± 131.38; *P* < 0.05) compared with those in the ND and HFD groups, indicating that the lack of carbohydrates and decreased amount of protein may have reduced intestinal microbial diversity in mice with obesity. Furthermore, the ND group had the highest abundance of intestinal species (4416), followed by the HFD and KD groups (3918 and 3762, respectively), and the HK group (2962), indicating that the combination of high-fat and ketogenic diets reduced the number of intestinal species (Fig. 2C).

Next, the mean abundance of gut bacteria was compared between four groups (Fig. 2D**)**. Bacteroidetes abundance was decreased in the HK group compared with that in the ND group. While the presence of Bacteroidetes can help maintain a healthy and stable intestinal tract, its decline in abundance is closely related to nervous system diseases, metabolic diseases, and tumors [26]. Verrucomicrobia abundance was increased in the HK and KD groups compared with that in the ND and HFD groups. The proportion of Firmicutes was increased in the HFD, HK, and KD groups compared with that in the ND group, but the difference was more significant in the HK group. Furthermore, Firmicutes emerged as the dominant phylum in the HK group, constituting 26% of all microbes, with the Firmicutes/Bacteroidetes ratio increasing from 56% in the HFD group to 247% in the HK group.

**Fig. 3:**
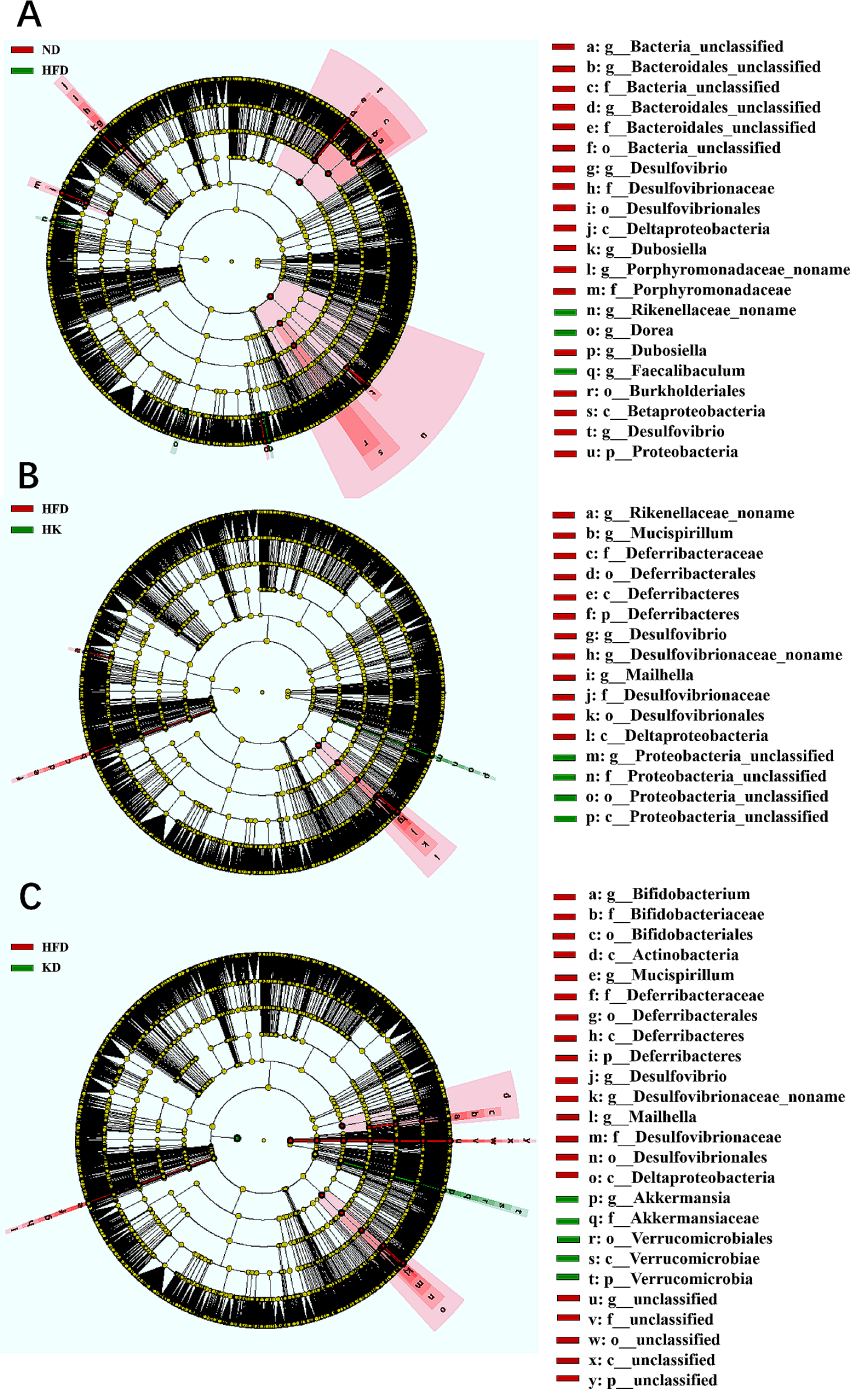
LEfSe generated cladogram to identify specific bacterial species in each group. **A**, specific bacterial species in ND and HFD group (*n* = 3); **B**, specific bacterial species in HK and HFD group (*n* = 3); **C**, specific bacterial species in KD and HFD group (*n* = 3)

The cladogram illustrates the classification levels variations in the four groups (Fig. 3 and Supplementary Figures S2–S4). The HFD group showed significantly higher abundances of the genera *Dorea (Bacillota*, anaerobic Gram+*)* and *Faecalibaculum (Bacillota*, anaerobic Gram+*)* than the ND group **(**Fig. 3A**)**. These genera have been implicated in obesity-associated dysbiosis [27–29]. The HK group exhibited a marked increase in the phylum Proteobacteria compared to the HFD group **(**Fig. 3B**)**. Elevated Proteobacteria levels indicate intestinal microbial imbalance or instability, which can trigger intestinal inflammation [30]. This finding suggested that the high-fat diet combined with a ketogenic feeding mode may compromise intestinal health. The KD group also displayed significant enrichment in class Verrucomicrobiae (Verrucomicrobiota, anaerobic Gram-), order Verrucomicrobiales (Verrucomicrobiota, anaerobic Gram-), family *Akkermansiaceae* (Verrucomicrobiota, anaerobic Gram-), and genus *Akkermansia* (Verrucomicrobiota, anaerobic Gram-) compared to the HFD group (Fig. 3C).

### Microbial functional alternations in the diet groups

**Fig. 4:**
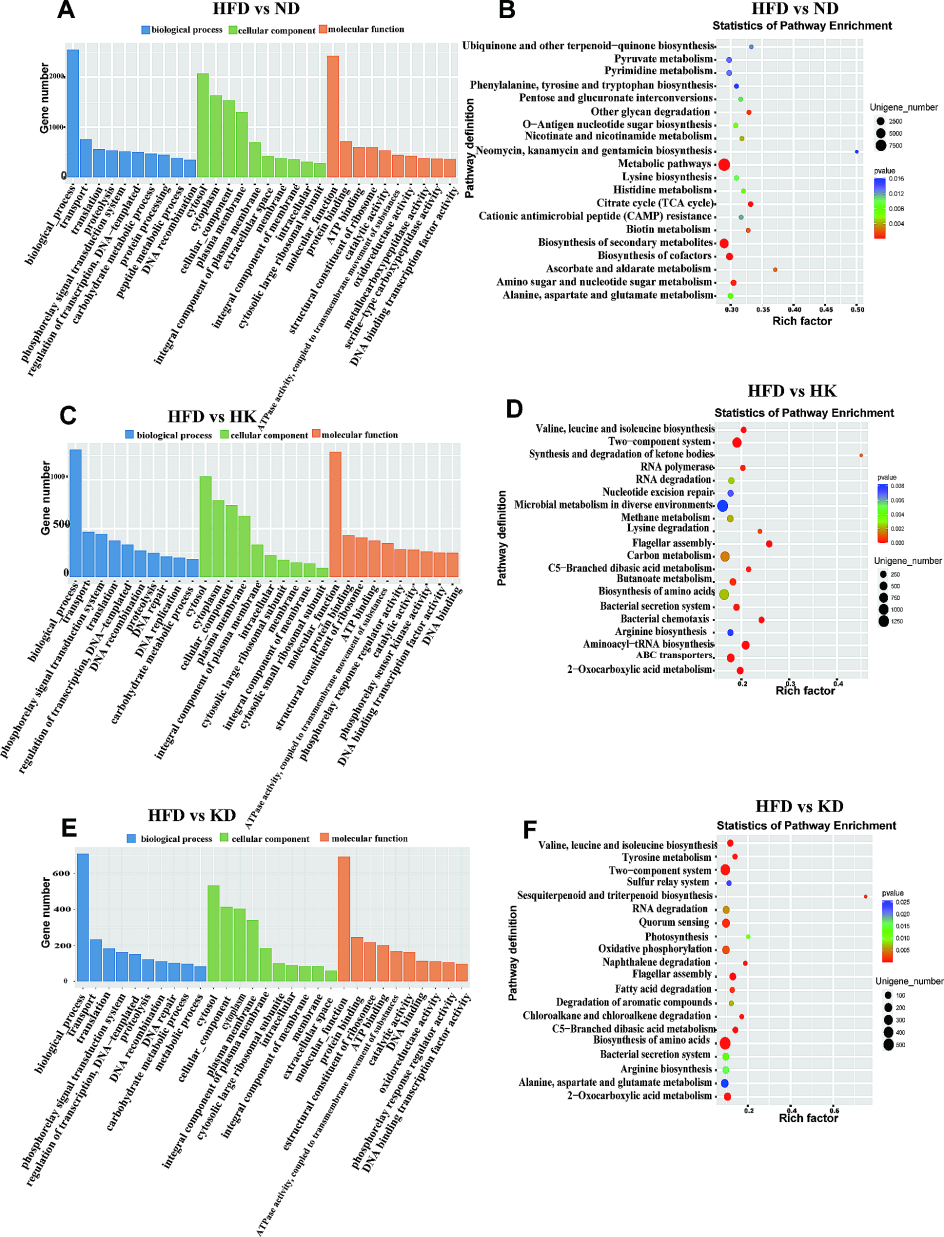
Enrichment of GO function and KEGG pathway of differentially expressed genes. **A**, the functions of differentially expressed genes between HFD and ND groups (*n* = 3); **B**, the altered metabolic pathways between HFD and ND groups (*n* = 3); **C**, the functions of differentially expressed genes between HFD and HK groups (*n* = 3); **D**, the altered metabolic pathways between HFD and HK groups (*n* = 3). **E**, the functions of differentially expressed genes between HFD and KD groups (*n* = 3); **F**, the altered metabolic pathways between HFD and KD groups (*n* = 3)

Genes with differential expression between the groups are shown in Supplementary Figure S5. The Gene Ontology (GO) and Kyoto Encyclopedia of Genes and Genomes (KEGG) databases were used to assess the functional implications of the microbial differences (Fig. 4 and Supplementary Figure S6). The functions of the changed genes between the HFD and ND groups are mainly linked to energy metabolism: ATP binding, ATPase activity, and metallocarboxypeptidase activity are related to ATP functions and protein degradation; transport/transmembrane transport of substances, translation, proteolysis, phosphorelay signal transduction system, and protein processing require energy expenditure; and oxidoreductase activity regulates the rate of redox reaction and is essential for most bioenergetic metabolic pathways (Fig. 4A). Furthermore, the high fat and low carbohydrate content of the high-fat diet also changed the carbohydrate metabolism process in the HFD group.

Metabolic pathways were altered between the HFD and ND groups (Fig. 4B), including amino acid synthesis and metabolism pathways comprising histidine, alanine, aspartate, and glutamate metabolism, phenylalanine, tyrosine, tryptophan, and lysine biosynthesis. Tryptophan synthesis in the gut occurs mainly through a metabolic pathway found in bacteria [31]. Changes in the tryptophan synthesis pathway may influence the types and functions of intestinal flora. Pathways related to sugar metabolism encompassed pentose and glucuronate interconversions, O-antigen nucleotide sugar biosynthesis, amino sugar and nucleotide sugar metabolism, and degradation of other glycans. Pathways of biosynthesis of cofactors and pyruvate metabolism were found to be connected to carbohydrate, energy, nucleotide, and amino acid metabolism. The citrate cycle, a central node of carbohydrate, lipid, and amino acid metabolism, was also significantly modified in the HFD group. Neomycin, kanamycin, and gentamicin biosynthesis were also affected in the HFD group. These changes in metabolic pathways indicates that the high-fat diet induced an imbalance of amino acid, carbohydrate, energy, and sugar metabolism.

Metabolic pathways were altered between the HFD and ND groups (Fig. 4B), including amino acid synthesis and metabolism pathways comprising histidine, alanine, aspartate, and glutamate metabolism, phenylalanine, tyrosine, tryptophan, and lysine biosynthesis. Tryptophan synthesis in the gut occurs mainly through a metabolic pathway found in bacteria [31]. Changes in the tryptophan synthesis pathway may influence the types and functions of intestinal flora. Pathways related to sugar metabolism encompassed pentose and glucuronate interconversions, O-antigen nucleotide sugar biosynthesis, amino sugar and nucleotide sugar metabolism, and degradation of other glycans. Pathways of biosynthesis of cofactors and pyruvate metabolism were found to be connected to carbohydrate, energy, nucleotide, and amino acid metabolism. The citrate cycle, a central node of carbohydrate, lipid, and amino acid metabolism, was also significantly modified in the HFD group. Neomycin, kanamycin, and gentamicin biosynthesis were also affected in the HFD group. These changes in metabolic pathways indicates that the high-fat diet induced an imbalance of amino acid, carbohydrate, energy, and sugar metabolism.

Next, the changes in functions and metabolic pathways between the HK and HFD groups were investigated. The functions of bacteria with abundance differences in the HFD and HK groups were found to be related to DNA binding, replication, recombination, and repair, as well as energy metabolism and expenditure (e.g., transport, translation, phosphorelay signal transduction system, proteolysis, protein binding, ATP binding, ATPase activity, and transmembrane movement of substances) (Fig. 4C). Notably, carbohydrate metabolic processes also showed differences between the HK and HFD groups, possibly because the proportion of carbohydrates was decreased from 20% in the high-fat diet to 0% in the ketogenic diet.

Altered metabolic pathways between the HK and HFD groups are shown in Fig. 4D. The ketogenic diet led to changes in amino acid metabolism, ABC transporters, lipid metabolism, carbon metabolism, ketone body metabolism, butanoate metabolism, methane metabolism, bacterial secretion and colonization (bacterial secretion system, flagella assembly, and bacterial chemotaxis), nucleotide synthesis, degradation, repair related pathways, two-component systems, 2-oxocarboxylic acid metabolism, and C5-branched dibasic acid metabolism in obese mice.

The functions and metabolic pathways that differed between the HFD and KD groups are shown in Fig. 4E and F, exhibiting most of the same changes in functions as those in the HFD and HK group comparison. Additional metabolic pathways that showed changes included tyrosine metabolism, sulfur relay system, sesquiterpenoid and triterpenoid biosynthesis, quorum sensing, photosynthesis, oxidative phosphorylation, degradation of naphthalene, fatty acid, chloroalkane, and chloroalkene, and aromatic compounds.

**Fig. 5:**
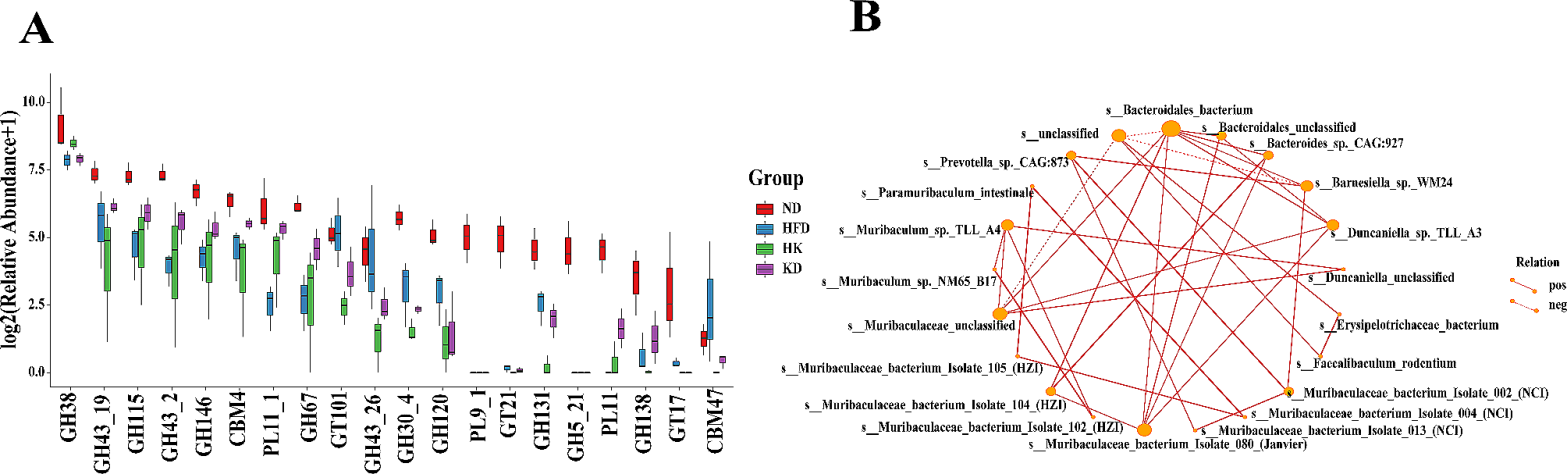
Carbohydrate-related enzymes abundance and dominant bacterias association maps between each group. **A**, Carbohydrate-related enzymes relative abundance between each group (*n* = 3); **B**, the correlation between dominant bacteria in four mice groups (*n* = 3); pos, positive correlation; neg, negative correlation. GH, Glycoside Hydrolases; GT, Glycosyl Transferases; CBM, Carbohydrate-Binding Module; PL, Polysaccharide Lyases; Data were calculated by the abundance and change relationship of different species in each mice group

The differences in carbohydrate-related enzymes between the four groups are shown in Fig. 5A (detailed in Supplementary Table 2). Except for glycosyltransferase (GT)101, glycoside hydrolases (GHs) 43_26, and carbohydrate-binding module (CBM) 47, most carbohydrate-related enzymes (e.g. GH38, GH43_19, GH115, GH43_2, GH146, CBM4, polysaccharide lyases (PLs)11_1, GH67, GH30_4, GH120, PL9_1, GT21, GH131, GH5_21, PL11, GH138, GT17) were increased in the ND group than in the HFD, HK, and KD groups. PL 9_1 and GH5_21 were not detected in HFD, HK, and KD groups. Compared with HFD group, GH115, GH43_2, GH146, PL 11_1, GH67, and PL11 showed significantly higher expression in the HK group. Eight enzymes, namely GH115, GH43_2, GH146, CBM4, PL11_1, GH67, PL11, and GH138 showed higher expression in the KD group than in the HFD group. Except for GH38, the expression of all remaining enzymes was higher in KD than in HK group.

Correlation between the two dominant bacterial groups was assessed on the basis of the abundances and altered relationships among different species in each group. The species that were related are shown in Fig. 5B. Bacteroidales (Bacteroidota, anaerobic Gram-) was the most abundant bacteria in the four groups, and was positively correlated with *Muribaculaceae* (Bacteroidota, anaerobic Gram-), *Duncaniella* (Bacteroidota, anaerobic Gram-), *Barnesiella* (Bacteroidota, anaerobic Gram-) and *Bacteroides* (Bacteroidota, anaerobic Gram-). *Muribaculaceae*, a family under Bacteroidales (Bacteroidota, anaerobic Gram-), was positively related to the genera *Duncaniella*, *Bacteroides*, *Paramuribaculum* (Bacteroidota, anaerobic Gram-), and *Muribaculum* (Bacteroidota, anaerobic Gram-). *Barnesiella* was positively correlated with Bacteroidales and *Prevotella* (Bacteroidota, anaerobic Gram-). *Faecalibaculum* was positively correlated with *Erysipelotrichaceaes (Bacillota, anaerobic Gram+*). Positive correlations were observed among families and genera within *Bacteroides*, as well as between the genera *Erysipelotrichaceaes* and *Faecalibaculum*. However, there were negative correlations among *Bacteroides*, *Erysipelotrichaceaes*, and *Faecalibaculum* that were related to the diet of the mice.

### Metabolic profiling of intestinal matter

**Fig. 6:**
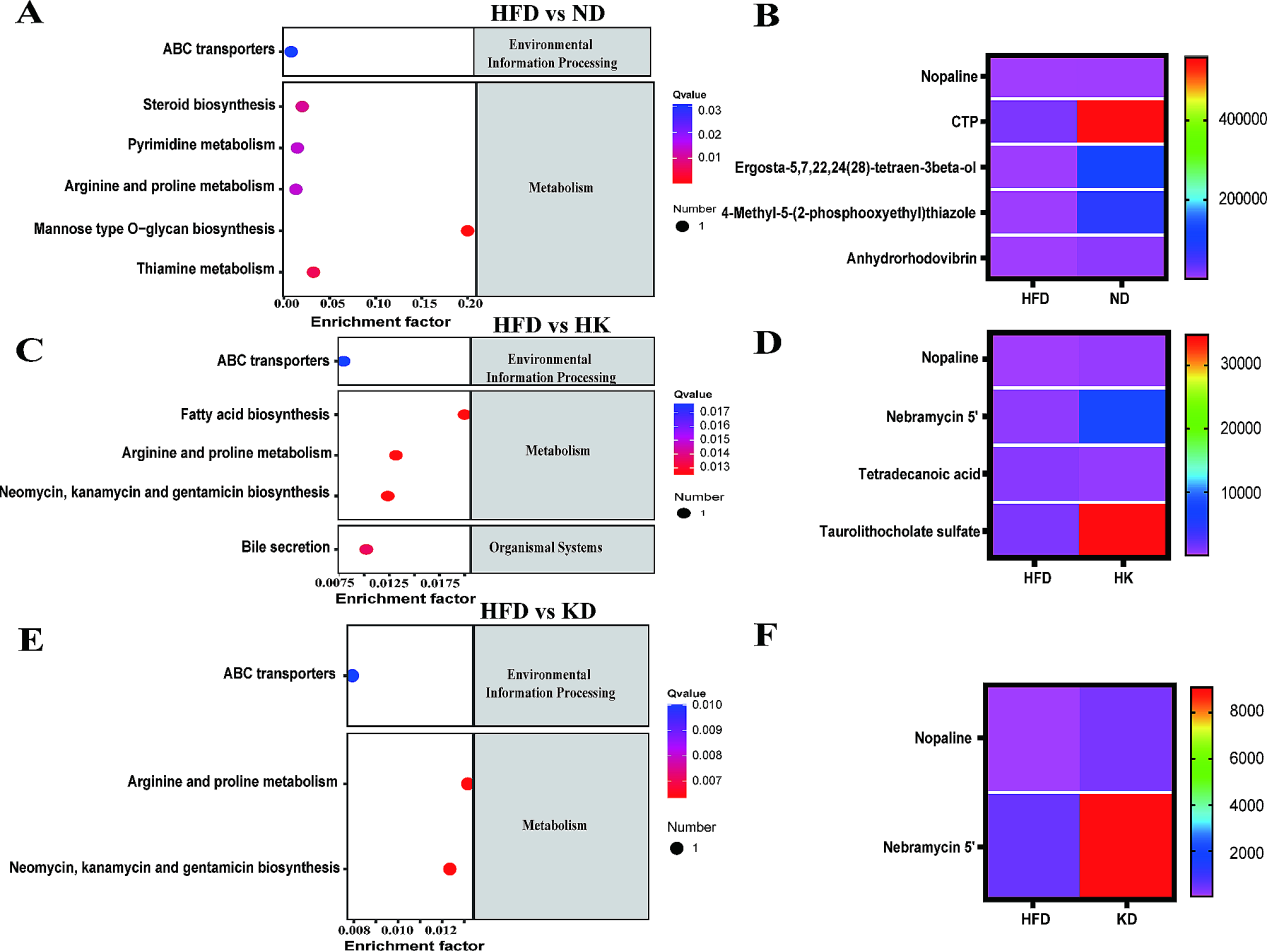
Differential metabolites and metabolic pathways between mice groups. **A**, metabolic pathways differed between the HFD and ND group (*n* = 6); **B**, heatmap showing the levels of the main metabolites associated with the metabolic pathways shown in A; **C**, metabolic pathways differed between the HK and HFD group (*n* = 6); **D**, heatmap showing the levels of the main metabolites associated with the metabolic pathways shown in C; E, metabolic pathways differed between the HFD and KD group (*n* = 6); F heatmap showing the levels of the main metabolites associated with the metabolic pathways shown in E

The intestinal contents of mice were analyzed using UPLC-MS/MS, and the metabolite spectrum was obtained by peak comparison and metabolite recognition. The metabolomics analysis identified 1594 different features at stage 2 of the UPLC-MS/MS (Supplementary Table 3) and quantified 903 metabolites (Supplementary Table 4). Most of the compounds in the KEGG pathway classification were related to protein digestion and absorption, amino acid metabolism and biosynthesis, glycerophospholipid metabolism, central carbon metabolism in cancer, bile secretion, and purine metabolism (Supplementary Figure S7). Thirty-two metabolites showed significant differences between the HFD and ND groups (Supplementary Figure S8). Five metabolites, namely nopaline, cytidine triphosphate, ergosta-5,7,22,24(28)-tetraen-3β-ol, 4-methyl-5-(2-phosphooxyethyl) thiazole, and anhydrorhodovibrin, were related to ABC transporters, steroid biosynthesis, pyrimidine, arginine, and proline metabolism, thiamine metabolism, and mannose type O-glycan biosynthesis (Fig. 6A and B).

Compared with HK and HFD groups, fifty-seven differential metabolites were identified (Supplementary Figure S9). Four of these — nopaline, nebramycin 5’, tetradecanoic acid, and taurolithocholate sulfate — were related to changes in pathways involving ABC transporters, fatty acid biosynthesis, arginine and proline metabolism, neomycin, kanamycin, and gentamicin biosynthesis, and bile secretion (Fig. 6C and D). 41 upregulated and downregulated metabolites were observed between the HFD group and KD group (Supplementary Figure S10). Two metabolites, nopaline, and nebramycin 5’, were related to the metabolic pathways involving ABC transporters, arginine and proline metabolism, and neomycin, kanamycin, and gentamicin biosynthesis (Fig. 6E and F).

## Discussion

This study explored the impacts of a ketogenic diet on body weight, fat mass, gut microbes, and metabolites in mice. The findings revealed a noteworthy decline in body weight, fat mass, and fat percentage in obese mice that were transitioned to a ketogenic diet, providing compelling evidence that a ketogenic diet is efficacious for shedding excess weight. Nevertheless, this investigation also showed that the ketogenic diet disrupted the high-fat diet-induced gut flora, potentially exacerbating the detrimental influences of the high-fat diet on the body.

As the study wore on, it became evident that the HFD group experienced marked elevations in body weight, fat mass, and fat percentage compared with the ND, HK, and KD groups. While a simple ketogenic diet did not lead to weight gain, there were slight increases in fat and fat percentage. However, HK group mice, which were switched to the ketogenic diet, experienced notable reductions in body weight, fat mass, and fat percentage compared to mice that remained on the high-fat diet. Collectively, these findings strongly indicated that the ketogenic diet effectively ameliorates obesity-related parameters, including body weight, body fat, and fat percentage.

Interestingly, microbial diversity and richness were diminished in the HK group, suggesting that the ketogenic diet impaired intestinal microbial diversity in obese mice. Firmicutes, Proteobacteria, and Verrucomicrobia were enriched, while Bacteroidetes was depleted, in the HK group, indicating that the ketogenic diet created an imbalance in gut microbes. Reduced Bacteroidetes abundance has been associated with intestinal homeostasis disruption, nervous system diseases, metabolic diseases, and tumors [26]. One study of Firmicutes and Bacteroidetes, two prominent phyla of the gut microbiota, demonstrated that a high-fat diet and a high-fiber diet led to higher abundances of Firmicutes and Bacteroidetes, respectively, in mice [32]. It has also been proposed that Firmicutes bacteria are more effective in absorbing energy compared with Bacteroidetes, thus promoting more efficient calorie absorption and the subsequent development of obesity and inflammatory diseases [33]. Therefore, the ratio of Firmicutes/Bacteroidetes has been widely used as an indicator to recognize normal intestinal homeostasis [34]. High Firmicutes/Bacteroidetes ratios have been linked to some pathological conditions, such as obesity and inflammation [35]. The disruption of gut homeostasis was also manifested by the elevated Firmicutes/Bacteroidetes ratio (247%) in the HK group, which has been associated with various pathological conditions and inflammation [34, 35]. Following the ketogenic diet intervention in this study, *Bacteroides* was negatively correlated with *Erysipelotrichaceaes*. *Bacteroides* are typically considered “lean bacteria,” whereas *Erysipelotrichaceaes* have been shown to be pro-inflammatory in host intestinal infection [36]. The LEfSe analysis revealed Proteobacteria to be a biomarker of the HK group. Proteobacteria is closely related to the imbalance or structural instability of the intestinal microbial community, reflecting intestinal inflammation [30]. Class Verrucomicrobiae and family *Akkermansiaceae* were identified as biomarkers of the KD group, with increased *Akkermansiaceae* being related to reduced body weight and fat mass in the KD group. Indeed, *Akkermansiaceae* has been previously negatively correlated with body weight and obesity [37].

In terms of microbial functions, the comparison between the HFD and ND groups showed that high-fat diet induced changes in host metabolism, manifesting as altered energy and carbohydrate metabolism. Furthermore, these changes were also involved in metabolic pathways, including tryptophan synthesis, sugar metabolism, citrate cycle, and aminoglycoside antibiotic biosynthesis (neomycin, kanamycin, and gentamicin). Compared with HFD group, the HK group displayed significant alterations in energy metabolism and expenditure-related functions. Significant differences were also detected in carbohydrate metabolic processes, which were correlated with the elimination of carbohydrates in the ketogenic diet.

The altered intestinal microbiota also contributes to butanoate (butyrate) metabolism, which is involved in maintaining the homeostasis of intestinal epithelial cells and regulating intestinal immune tolerance to antigens [38]. Altered butanoate metabolism is also related to the aggravation of inflammatory reactions and immune dysfunction [39]. Methane, a gas produced by gut bacteria, slows intestinal transit and enhances the contractile activity of the small intestine [40]. Microbial methane metabolism is a potential regulator of host energy balance, thus changes in methane metabolism may indicate disruption in this balance [41]. The KEGG analysis showed enrichment of pathways related to bacterial secretion and colonization, such as bacterial secretion systems, flagella assembly, and bacterial chemotaxis, in the HK group, indicating that the ketogenic diet changed the species and quantity of intestinal microorganisms in obese mice. Nucleotide synthesis, degradation, repair, and other pathways related to intestinal inflammation and body immunity, including RNA degradation, RNA polymerase, nucleotide excision repair, and aminoacyl-tRNA biosynthesis, also significantly changed in the HK group. Two-component systems, which are mechanisms by which bacteria adapt to selective pressure, also showed significant changes in obese mice under the ketogenic diet, presumably as a means to alleviate stress. Metabolism of 2-oxocarboxylic acid, which is associated with energy and lipid metabolism, was greatly enriched in HK mice compared with that in HFD mice. Such changes in C5-branched dibasic acid metabolism can affect lipid metabolism through lipase and acetolactate synthase [42].

Expression of almost all carbohydrate-related enzymes were higher in the ND group (e.g. GH38, GH43_19, GH115, GH43_2, GH146, CBM4, PL11_1, GH67, GH30_4, GH120, PL9_1, GT21, GH131, GH5_21, PL11, GH138, GT17), indicating that carbohydrate intake in the normal diet (65% carbohydrate, 18% protein, 17% fat) increased the expression of related enzymes. Generally, the expression of carbohydrate-related enzymes decreased in mice with HFD, HK, and KD groups. PL9-1, an enzyme that cleaves polysaccharide chains to generate unsaturated hexenuronic acid residue, and GH5-21, a GH that hydrolyzes glycosidic bonds between carbohydrates, were not detected in the HFD, HK, and KD groups, suggesting that these enzymes are not expressed in a high-fat or ketogenic diet state. Because both PL9-1 and GH5-21 are involved in the hydrolysis of carbohydrates including polysaccharides, the loss of microbial function associated with these enzymes suggests that reduced carbohydrate intake leads to substrate deficiency. However, obese and ketogenic diet-fed mice often exhibit inflammation and gut microbiota disorder, so reductions in PL9-1- and GH5-21-associated microbes may have potential as future biomarkers for gut microbiota disorder. Compared to HFD group, the expression of nearly half of the 20 enzymes involved in carbohydrate increased in the HK and KD groups. This may be attributable to a compensatory bodily reaction to the absence of carbohydrates, or may be associated with inflammation.

Among the metabolomics results, the upregulated bile secretion (taurolithocholate sulfate; Fig. 6D) in the HK group indicated that the high-fat content of the ketogenic diet increases the relative difficulty of fat digestion, making it necessary to adapt by secreting more bile to promote fat digestion, absorption, and intestinal peristalsis. Bile acids, produced in the liver and metabolized by enzymes from intestinal bacteria, are crucial for maintaining gut microbiota, innate immunity, insulin sensitivity, and the metabolism of lipids and carbohydrates [43]. Nevertheless, the decrease in gut microbiota abundance in HK mice may have led to decreased bile acid metabolism. Fatty acids serve as primary energy sources, and a high-fat ketogenic diet results in elevated levels of palmitoyl CoA within cells. This feedback inhibits acetyl CoA carboxylase, thereby inhibiting the synthesis of fatty acids. Therefore, fatty acid synthesis was downregulated in the HK group. The metabolomics analysis also showed upregulation of the neomycin, kanamycin, and gentamicin biosynthesis pathways in the HK and KD group. Indeed, these aminoglycosides have been reported to reduce the number of species and increase the proportions of Firmicutes, Proteobacteria, and Verrucomicrobia in the gut microbiota [44, 45]. Hence, a high-fat ketogenic diet boosts aminoglycoside antibiotic synthesis in the intestine, reducing beneficial gut microbes and increasing pathogenic species of Firmicutes and Proteobacteria. Additionally, *Akkermansiaceae*, a family within Verrucomicrobia, is reportedly inversely correlated with body weight [46]. Changes in the composition and abundance of intestinal flora lead to further dysfunction of the gut microbiota.

### Strengths

This study provides a comprehensive analysis of the impact of a ketogenic diet on obesity, utilizing both metagenomic and metabolomic approaches. By employing high-precision measurements and advanced analytical techniques, the research offers detailed insights into how a ketogenic diet can influence body composition, gut microbiota diversity, and metabolic pathways in obese mice. Significant findings, including the reduction in microbial diversity and alterations in metabolic pathways, highlight the complex interactions between diet and gut health. These insights are crucial for understanding the potential adverse effects of ketogenic diets, contributing valuable knowledge to the field of nutritional science and obesity management.

### Limitations

The mouse model used in this study may not fully reflect the effects of a ketogenic diet on the human gut microbiota and metabolism. The effect of the low protein level of the ketogenic diet on the gut requires further research. Analysis of lipid metabolites may increase the significance of this study. Importantly, data on mouse activity, caloric excretion in feces, and caloric bomb measurements of short-chain fatty acids in cecal content were not collected. These omissions may limit the ability to fully elucidate the dynamics of dietary intake, metabolic responses, and gut microbiota composition in the context of ketogenic diet intervention. Future studies incorporating these measurements are warranted to provide a more holistic understanding of the metabolic mechanisms underlying the observed outcomes. Additionally, further studies are needed to validate the findings in human subjects.

## Conclusions

This study provides evidence that a ketogenic diet can effectively decrease body weight, fat mass, and fat percentage in obese mice. However, it is important to note that this diet also had adverse effects on intestinal microbial diversity and balance, inducing alterations in several metabolic pathways, including those related to energy, carbohydrates, lipids, amino acids, and nucleotides, as well as bacterial secretion and colonization. These changes suggest a potential increase in intestinal inflammation and disruptions in defense mechanisms when using a low-carbohydrate diet to treat obesity. These findings also emphasize the clinical significance of understanding the interplay between diet, gut microbiota, and metabolic health, highlighting the importance of personalized dietary interventions tailored to individual gut microbiome profiles for patients with obesity and metabolic disorders. Monitoring changes in gut microbiota composition and metabolic pathways as biomarkers of metabolic health and disease risk may be valuable for guiding personalized interventions. Furthermore, identification of specific microbial alterations and metabolic pathways associated with dietary interventions like the ketogenic diet provides insights into potential therapeutic targets for managing obesity and related metabolic disorders. Looking ahead, longitudinal studies and precision medicine approaches that integrate multiomics data are necessary to translate these findings into tangible benefits for patients, thereby advancing patient care and outcomes in the field of metabolic medicine.

### Electronic supplementary material

Below is the link to the electronic supplementary material.

Supplementary Material 1

Supplementary Material 2

Supplementary Material 3

Supplementary Material 4

Supplementary Material 5

Supplementary Material 6

Supplementary Material 7

Supplementary Material 8

Supplementary Material 9

Supplementary Material 10

Supplementary Material 11

Supplementary Material 12

Supplementary Material 13

Supplementary Material 14

Supplementary Material 15

## Data Availability

The original data presented in this study can be found online at: https://www.ncbi.nlm.nih.gov/bioproject/PRJNA956273. For further requirements, please contact the corresponding author.

## Abbreviations

CBM: Carbohydrate-Binding Module
GT: Glycosyl Transferases
GHs: Glycoside Hydrolases
GO: Gene Ontology
HFD: High-fat diet
HK: HFD converted to a ketogenic diet
IDA: Information dependent acquisition
KD: Ketogenic diet
KEGG: Kyoto Encyclopedia of Genes and Genomes
LEfSe: Linear discriminant analysis Effect Size
UPLC-MS/MS: Ultra-performance liquid chromatography-tandem mass spectrometry
ND: Normal diet
PCA: Principal Component Analysis
PLS-DA: Partial least Squares-Discriminant Analysis
PL: Polysaccharide Lyases
QC: Quality control
VIP: Variable importance in projection
