# Supplementary material for: Metagenomic and metabolomic analysis showing the adverse risk–benefit trade-off of the ketogenic diet
Source: Lipids Health Dis. 2024 Jun 29;23:207. doi: 10.1186/s12944-024-02198-7 (PMC11218088; doi:10.1186/s12944-024-02198-7)
Supplement: Supplementary file 1 — Supplementary Material 1 [file 12944_2024_2198_MOESM1_ESM.docx]

| **Table S1,**  **precise visualization of the data shown in Figure1** | | | | |
| --- | --- | --- | --- | --- |
| **Group** | **Week** | **Weight(g)** | **Body fat(g)** | **Fat/Weight(%)** |
| **ND** | **0** | 15.90 16.00 13.60 16.50 15.80 16.60 15.70 15.30 | 0.80 1.21 0.88 1.30 0.78 1.28 1.56 1.29 | 5.03 7.58 6.50 7.85 4.91 7.72 9.94 8.45 |
|  |  | 21.70 18.90 19.70 18.90 18.40 21.70 18.10 18.40 | 1.48 0.48 1.82 1.55 1.61 2.21 1.57 1.26 | 6.84 2.53 9.24 8.18 8.73 10.16 8.69 6.83 |
|  |  | 23.30 20.20 20.00 20.20 19.60 23.20 19.50 20.00 | 1.46 0.85 0.72 1.60 1.52 1.55 1.48 1.19 | 6.25 4.19 3.58 7.94 7.75 6.68 7.60 5.95 |
|  |  | 24.50 21.70 21.20 21.30 20.80 25.00 20.20 21.10 | 1.44 1.01 0.90 1.67 1.96 1.81 1.95 1.65 | 5.87 4.66 4.24 7.85 9.42 7.25 9.64 7.82 |
|  |  | 25.40 22.70 23.10 21.70 20.50 25.80 21.50 21.90 | 1.90 1.21 1.21 1.87 1.68 1.53 1.70 1.42 | 7.46 5.32 5.23 8.61 8.18 5.91 7.92 6.48 |
|  |  | 26.20 23.40 24.30 22.40 21.70 26.80 21.80 23.00 | 1.50 1.83 1.48 1.69 1.86 1.87 2.11 1.90 | 5.71 7.80 6.07 7.54 8.55 6.99 9.66 8.27 |
|  |  | 26.30 23.40 24.30 22.40 21.80 27.30 22.00 23.10 | 1.99 1.94 1.69 1.84 1.98 1.72 1.65 1.90 | 7.58 8.27 6.94 8.19 9.10 6.30 7.51 8.21 |
|  |  | 26.20 23.70 24.50 22.40 22.10 28.50 22.50 23.00 | 1.97 1.63 1.16 1.41 1.66 1.43 1.73 1.38 | 7.52 6.87 4.74 6.24 7.53 5.01 7.68 5.98 |
|  |  | 26.40 23.40 25.60 22.40 22.90 28.70 22.90 23.50 | 2.19 2.03 1.90 1.96 2.09 2.06 2.12 2.02 | 8.30 8.65 7.42 8.43 9.12 7.18 9.26 8.60 |
|  |  | 26.60 23.90 25.40 22.40 23.20 28.90 24.40 25.40 | 2.21 2.14 1.75 2.19 2.51 1.80 2.32 2.43 | 8.29 8.95 6.90 9.19 10.82 6.24 9.51 9.58 |
|  |  | 27.30 24.10 25.30 22.40 23.00 30.00 23.60 24.40 | 2.28 2.10 1.76 2.01 2.10 1.93 1.95 1.65 | 8.36 8.70 6.96 8.71 9.13 6.42 8.28 6.75 |
|  |  | 27.60 24.60 26.10 22.40 24.20 29.70 23.70 24.30 | 2.22 2.52 2.25 1.86 2.39 1.91 1.51 1.62 | 8.04 10.22 8.63 7.77 9.89 6.43 6.37 6.67 |
|  |  | 27.70 24.30 24.20 22.40 24.00 31.30 24.70 25.40 | 2.63 1.78 1.89 2.18 2.20 2.43 2.11 1.91 | 9.48 7.33 7.82 8.89 9.15 7.77 8.53 7.50 |
|  |  | 27.40 25.60 25.80 22.40 23.40 30.00 24.00 25.70 | 2.18 2.65 2.00 1.99 2.00 2.16 1.89 1.80 | 7.94 10.35 7.74 8.41 8.55 7.19 7.89 7.01 |
|  |  | 28.20 24.70 25.00 22.40 23.70 30.00 24.30 25.20 | 2.82 2.03 1.96 2.22 2.35 2.39 1.96 1.99 | 9.99 8.22 7.85 9.01 9.89 7.96 8.06 7.91 |
|  |  | 28.40 25.70 26.50 22.40 24.10 30.00 24.60 25.00 | 2.87 2.77 1.79 2.36 2.46 2.14 2.23 1.86 | 10.10 10.80 6.74 9.45 10.21 7.13 9.05 7.45 |
|  |  | 28.80 25.90 26.90 22.40 24.00 30.60 25.00 26.50 | 2.70 2.92 2.10 1.97 2.41 1.98 2.21 1.98 | 9.39 11.28 7.81 7.93 10.06 6.47 8.83 7.46 |
|  |  | 28.00 26.00 26.70 22.40 23.70 30.00 23.70 25.20 | 2.28 2.59 1.93 1.89 2.50 1.22 1.79 2.08 | 8.13 9.96 7.23 7.75 10.56 4.06 7.56 8.27 |
|  |  | 28.40 25.90 27.60 22.40 24.00 31.20 24.50 26.60 | 2.36 2.56 2.02 2.35 2.38 2.30 1.92 1.94 | 8.31 9.89 7.33 9.42 9.93 7.36 7.85 7.28 |
|  |  | 29.50 26.90 28.00 22.40 23.90 31.60 25.30 26.80 | 2.73 2.48 2.16 2.36 2.43 2.11 2.04 2.31 | 9.25 9.20 7.72 9.52 10.18 6.68 8.04 8.64 |
|  |  | 29.20 26.70 28.10 22.40 24.60 31.30 25.70 26.80 | 2.34 2.36 2.43 2.17 2.51 2.50 1.94 2.09 | 8.02 8.82 8.63 8.42 10.20 8.00 7.55 7.82 |
| **HF** | **0** | 14.70 13.60 14.10 14.00 15.80 16.40 15.80 16.10 | 1.18 1.21 1.29 1.11 1.06 1.06 1.15 1.19 | 8.01 8.87 9.11 7.92 6.68 6.48 7.25 7.41 |
|  | **1** | 21.70 20.50 20.70 21.10 22.10 22.20 21.00 20.00 | 2.71 1.91 1.43 1.86 1.71 1.90 1.35 1.79 | 12.51 9.34 6.92 8.80 7.72 8.57 6.43 8.93 |
|  | **2** | 23.10 21.80 22.50 22.90 23.50 23.30 22.40 21.40 | 2.76 2.08 2.06 1.80 1.66 1.74 1.26 1.72 | 11.93 9.53 9.16 7.87 7.06 7.48 5.60 8.03 |
|  | **3** | 25.40 23.20 23.00 23.80 25.90 25.20 23.80 21.80 | 2.79 2.44 2.23 2.09 1.58 2.10 1.05 1.82 | 10.99 10.52 9.68 8.78 6.10 8.35 4.42 8.33 |
|  | **4** | 26.80 23.40 24.40 25.40 26.80 24.80 24.70 23.40 | 2.68 2.42 2.38 2.06 1.43 1.62 1.13 1.88 | 10.00 10.35 9.76 8.12 5.34 6.53 4.59 8.04 |
|  | **5** | 28.10 25.90 26.90 25.80 28.30 27.90 26.50 23.80 | 3.29 3.51 3.48 2.02 2.07 2.52 1.05 1.98 | 11.69 13.56 12.93 7.83 7.33 9.03 3.97 8.33 |
|  | **6** | 30.80 26.70 28.70 26.80 30.30 29.40 27.30 25.20 | 4.31 3.96 4.48 2.29 2.74 3.42 1.26 2.25 | 13.99 14.81 15.62 8.54 9.06 11.64 4.62 8.91 |
|  | **7** | 31.40 27.30 28.80 27.30 32.30 29.60 27.70 25.20 | 4.68 4.15 4.52 2.14 3.42 3.87 1.82 2.44 | 14.92 15.21 15.68 7.85 10.60 13.07 6.57 9.67 |
|  | **8** | 32.50 29.00 30.30 28.20 32.70 31.20 28.50 26.10 | 5.38 5.34 5.13 3.12 3.54 4.21 1.96 2.56 | 16.56 18.42 16.94 11.05 10.84 13.48 6.87 9.82 |
|  | **9** | 34.00 30.60 32.10 28.30 33.80 32.50 29.60 27.00 | 6.20 5.90 5.91 2.71 4.07 4.73 1.82 2.92 | 18.24 19.27 18.41 9.59 12.04 14.56 6.16 10.81 |
|  | **10** | 34.70 31.10 32.30 28.10 34.50 33.30 29.40 27.10 | 6.67 6.03 5.98 2.59 4.48 5.07 2.03 2.71 | 19.23 19.38 18.51 9.22 12.97 15.23 6.91 10.01 |
|  | **11** | 34.60 31.00 32.80 28.10 35.30 33.70 30.80 27.40 | 6.52 6.28 6.15 2.62 4.67 5.43 2.48 3.01 | 18.83 20.27 18.74 9.34 13.23 16.12 8.05 10.98 |
|  | **12** | 36.30 33.90 34.50 28.90 34.50 35.10 30.20 28.70 | 7.00 7.53 6.96 2.97 3.64 5.29 1.78 3.54 | 19.28 22.20 20.18 10.27 10.56 15.08 5.88 12.32 |
|  | **13** | 37.20 34.10 35.10 30.00 34.20 35.50 30.30 28.50 | 7.62 8.02 7.38 3.23 3.45 5.68 1.75 3.38 | 20.49 23.51 21.03 10.76 10.07 15.99 5.77 11.86 |
|  | **14** | 38.10 34.30 35.10 29.50 34.80 36.30 30.80 28.50 | 8.46 8.36 7.78 3.28 3.92 5.96 1.71 3.23 | 22.19 24.37 22.16 11.11 11.26 16.41 5.56 11.33 |
|  | **15** | 38.80 35.00 36.80 29.80 34.50 35.70 30.00 29.00 | 9.03 8.63 8.56 3.59 3.99 6.52 1.99 3.71 | 23.26 24.67 23.25 12.04 11.56 18.26 6.62 12.80 |
|  | **16** | 39.40 34.90 36.40 29.40 36.70 37.90 30.60 29.80 | 9.32 8.73 8.72 3.22 5.26 7.56 2.52 3.90 | 23.64 25.02 23.96 10.96 14.33 19.94 8.24 13.07 |
|  | **17** | 39.60 35.00 36.80 29.60 36.10 37.00 29.30 30.00 | 9.32 8.33 8.69 2.84 4.71 7.12 1.79 3.69 | 23.53 23.81 23.62 9.58 13.05 19.23 6.11 12.30 |
|  | **18** | 40.20 35.30 38.20 29.40 36.70 37.30 30.00 29.80 | 9.97 8.78 9.37 4.14 5.91 7.40 2.15 4.04 | 24.81 24.87 24.52 14.07 16.10 19.83 7.17 13.54 |
|  | **19** | 41.40 36.70 39.60 30.40 38.20 38.80 30.60 30.70 | 10.41 9.65 10.30 3.57 6.85 8.32 2.62 4.40 | 25.14 26.30 26.00 11.73 17.94 21.44 8.57 14.33 |
|  | **20** | 42.00 38.30 40.80 30.40 39.40 39.60 30.20 31.10 | 10.86 10.38 10.77 4.00 7.66 8.91 2.74 4.57 | 25.86 27.11 26.40 13.15 19.43 22.50 9.08 14.69 |
| **HK** | **0** | 15.70 16.70 17.00 18.10 15.40 16.00 15.20 16.40 | 1.25 1.11 0.95 1.55 1.06 1.35 1.25 1.24 | 7.94 6.63 5.58 8.56 6.90 8.41 8.24 7.55 |
|  | **1** | 20.30 20.30 20.80 24.40 20.00 21.60 20.00 21.00 | 1.49 1.16 2.23 2.42 1.60 1.83 1.72 1.73 | 7.36 5.72 10.70 9.93 7.98 8.45 8.60 8.22 |
|  | **2** | 22.00 22.00 22.30 25.70 21.60 23.20 21.10 22.50 | 1.68 1.49 2.09 2.08 1.99 1.83 2.06 1.99 | 7.65 6.78 9.39 8.11 9.21 7.90 9.78 8.86 |
|  | **3** | 23.20 22.50 23.90 27.30 22.60 25.40 21.50 23.80 | 1.57 1.57 2.43 1.76 1.83 2.30 2.29 2.05 | 6.75 7.00 10.17 6.44 8.12 9.05 10.63 8.59 |
|  | **4** | 24.80 24.20 25.30 28.50 24.00 26.80 23.00 25.50 | 1.43 1.75 2.08 1.70 1.72 2.43 2.65 2.31 | 5.76 7.21 8.23 5.96 7.18 9.07 11.50 9.06 |
|  | **5** | 25.40 25.80 27.10 29.70 25.00 27.90 23.50 26.30 | 1.55 1.82 2.03 1.71 1.78 3.01 2.66 2.83 | 6.08 7.06 7.51 5.75 7.11 10.77 11.32 10.77 |
|  | **6** | 26.40 27.20 28.90 31.30 27.00 30.80 24.20 27.60 | 1.70 2.08 3.67 2.14 2.54 4.35 3.10 3.39 | 6.45 7.64 12.70 6.85 9.40 14.13 12.81 12.27 |
|  | **7** | 26.80 26.80 30.00 32.30 26.90 32.00 24.50 28.80 | 1.62 1.63 4.57 2.64 2.61 4.80 3.10 3.44 | 6.06 6.08 15.24 8.18 9.70 14.99 12.65 11.94 |
|  | **8** | 26.90 27.80 31.60 33.00 28.10 33.80 25.80 29.30 | 1.41 1.69 5.21 3.31 3.21 6.10 3.61 3.80 | 5.22 6.07 16.50 10.03 11.42 18.04 13.99 12.98 |
|  | **9** | 27.80 27.90 32.90 35.10 29.40 36.50 27.30 30.20 | 1.79 1.79 6.10 4.56 4.03 7.51 4.13 4.13 | 6.44 6.42 18.54 12.99 13.70 20.58 15.14 13.68 |
|  | **10** | 27.60 28.60 32.40 35.30 29.40 37.40 27.60 31.00 | 1.59 1.61 5.59 4.26 3.69 8.12 4.14 4.21 | 5.76 5.64 17.26 12.06 12.56 21.72 15.01 13.56 |
|  | **11** | 28.00 29.10 31.80 35.40 29.60 38.80 28.00 31.80 | 1.96 1.98 5.49 4.76 3.78 9.13 4.55 4.61 | 6.99 6.80 17.28 13.44 12.78 23.53 16.25 14.51 |
|  | **12** | 30.00 30.50 34.40 37.80 31.90 37.50 29.60 33.20 | 2.34 2.52 5.55 5.41 4.71 7.49 4.82 5.07 | 7.80 8.25 16.13 14.31 14.76 19.98 16.30 15.26 |
|  | **13** | 25.60 27.50 27.70 31.30 26.70 30.60 25.10 27.50 | 1.59 1.65 2.60 3.04 2.87 4.19 3.04 3.17 | 6.20 5.98 9.39 9.72 10.76 13.69 12.10 11.54 |
|  | **14** | 25.90 27.70 28.90 32.40 27.90 28.80 23.90 27.50 | 1.90 2.12 3.24 3.48 3.80 3.11 2.79 3.35 | 7.34 7.64 11.22 10.73 13.62 10.78 11.66 12.18 |
|  | **15** | 27.00 28.20 28.20 32.60 28.10 29.10 24.20 28.30 | 2.66 2.13 2.75 3.28 3.40 3.11 2.95 3.53 | 9.86 7.57 9.74 10.07 12.11 10.68 12.21 12.48 |
|  | **16** | 26.60 27.50 28.90 32.40 27.70 29.40 24.30 29.30 | 2.21 2.25 3.34 3.47 3.60 3.47 2.85 3.41 | 8.32 8.17 11.57 10.72 13.00 11.79 11.73 11.63 |
|  | **17** | 26.90 28.20 28.40 32.70 27.90 29.30 25.50 29.00 | 2.43 2.38 2.71 3.33 3.34 3.00 3.10 3.51 | 9.01 8.43 9.53 10.18 11.98 10.24 12.15 12.09 |
|  | **18** | 26.70 27.30 28.70 33.50 28.40 30.30 25.40 29.40 | 2.07 2.13 3.11 3.07 3.97 3.62 3.14 3.82 | 7.76 7.81 10.85 9.16 14.00 11.96 12.37 13.01 |
|  | **19** | 26.20 27.30 28.80 33.50 28.60 30.40 25.20 29.40 | 2.08 2.07 3.09 4.30 4.02 4.11 2.96 3.76 | 7.93 7.57 10.73 12.83 14.04 13.51 11.73 12.79 |
|  | **20** | 26.30 27.30 29.20 34.10 28.70 30.50 25.60 29.60 | 2.17 1.99 3.48 4.61 4.34 4.11 3.30 3.89 | 8.27 7.30 11.92 13.51 15.14 13.46 12.87 13.13 |
| **KD** | **0** | 16.40 14.60 16.70 15.90 17.30 15.80 16.10 16.80 | 1.10 1.10 1.40 1.49 1.03 1.15 1.11 0.99 | 6.70 7.51 8.41 9.37 5.96 7.25 6.89 5.89 |
|  | **1** | 17.00 15.80 18.00 18.10 18.90 18.60 17.50 17.60 | 2.08 1.37 2.02 1.97 2.20 1.96 1.54 1.83 | 12.25 8.66 11.19 10.87 11.62 10.56 8.80 10.42 |
|  | **2** | 17.90 16.30 18.60 18.40 19.40 18.10 18.20 18.40 | 2.18 2.00 2.68 2.49 2.50 2.41 1.95 1.89 | 12.17 12.25 14.40 13.53 12.89 13.29 10.73 10.29 |
|  | **3** | 20.20 17.70 19.80 20.60 20.60 20.20 20.30 20.20 | 2.20 1.45 1.97 2.28 2.31 2.57 2.20 2.27 | 10.87 8.20 9.94 11.05 11.20 12.74 10.83 11.25 |
|  | **4** | 21.30 18.90 20.90 22.00 22.00 20.70 21.40 19.60 | 2.28 1.79 2.49 2.38 1.95 2.12 1.87 2.17 | 10.69 9.46 11.90 10.82 8.85 10.22 8.72 11.08 |
|  | **5** | 21.70 19.60 21.70 22.60 22.60 21.80 22.30 21.90 | 1.89 1.89 2.44 2.11 2.64 2.47 2.01 2.23 | 8.73 9.63 11.24 9.35 11.66 11.34 9.00 10.17 |
|  | **6** | 23.10 20.30 22.00 24.30 23.60 22.30 22.80 23.00 | 2.22 1.92 2.33 2.38 2.83 2.69 1.96 2.58 | 9.61 9.47 10.60 9.80 12.00 12.04 8.60 11.22 |
|  | **7** | 22.50 20.00 22.40 24.70 23.30 22.70 22.70 22.90 | 2.06 1.71 2.31 1.89 2.38 2.11 1.54 1.98 | 9.14 8.54 10.31 7.66 10.22 9.28 6.78 8.65 |
|  | **8** | 23.00 20.60 23.70 25.10 24.00 23.00 23.30 23.10 | 2.58 2.48 3.03 2.72 2.99 2.59 2.11 2.08 | 11.22 12.05 12.79 10.84 12.44 11.27 9.03 8.99 |
|  | **9** | 23.40 20.90 23.90 25.20 23.80 23.70 21.70 23.50 | 2.58 2.08 2.70 2.56 3.21 2.91 2.30 2.50 | 11.03 9.93 11.28 10.14 13.49 12.26 10.58 10.65 |
|  | **10** | 23.80 20.70 23.60 25.30 23.70 23.80 23.20 23.70 | 2.67 2.18 2.47 2.52 2.43 2.96 1.86 2.51 | 11.21 10.53 10.48 9.96 10.25 12.43 8.03 10.59 |
|  | **11** | 23.90 21.10 24.10 25.50 24.20 23.30 23.50 23.80 | 2.53 2.00 2.74 2.41 2.49 2.69 2.33 2.42 | 10.58 9.46 11.38 9.43 10.30 11.53 9.92 10.16 |
|  | **12** | 24.40 21.80 24.90 26.30 24.20 24.10 24.00 24.20 | 2.96 2.36 2.99 2.89 2.56 2.83 2.08 2.50 | 12.11 10.81 11.99 11.00 10.58 11.76 8.66 10.35 |
|  | **13** | 24.70 21.70 25.20 25.90 23.80 23.80 23.70 24.70 | 2.71 2.39 3.02 2.98 2.68 2.32 2.02 2.48 | 10.98 11.00 11.96 11.49 11.27 9.76 8.50 10.05 |
|  | **14** | 24.20 21.00 24.50 26.40 24.50 23.80 23.10 23.80 | 2.96 2.18 2.97 2.84 2.71 2.57 2.20 2.51 | 12.23 10.36 12.12 10.77 11.07 10.78 9.53 10.56 |
|  | **15** | 24.70 21.50 25.10 26.10 25.10 24.20 23.20 24.70 | 2.80 2.38 3.41 3.11 2.85 2.40 2.08 2.67 | 11.35 11.06 13.60 11.92 11.36 9.94 8.98 10.83 |
|  | **16** | 24.60 21.70 25.30 26.00 25.40 23.90 23.70 25.00 | 2.52 2.14 2.78 2.70 2.77 2.62 2.42 3.24 | 10.24 9.86 10.99 10.39 10.89 10.95 10.22 12.95 |
|  | **17** | 24.60 22.10 25.80 25.90 25.20 24.20 24.20 25.80 | 2.90 2.29 3.08 2.74 3.04 2.54 2.39 2.90 | 11.78 10.37 11.94 10.58 12.08 10.51 9.89 11.25 |
|  | **18** | 25.10 22.60 25.30 25.90 25.30 24.10 23.50 26.00 | 2.84 2.79 2.95 3.04 3.08 2.60 2.41 2.88 | 11.31 12.34 11.64 11.73 12.19 10.79 10.27 11.08 |
|  | **19** | 25.40 22.60 25.50 26.20 25.50 24.90 24.20 26.90 | 2.83 2.92 3.13 2.91 2.94 2.86 2.68 3.20 | 11.15 12.94 12.27 11.09 11.53 11.47 11.07 11.91 |
|  | **20** | 25.60 22.90 25.60 26.40 25.40 25.40 23.80 27.90 | 3.16 3.29 3.33 3.03 3.05 2.52 2.72 4.00 | 12.33 14.37 12.99 11.47 12.00 9.93 11.45 14.33 |
