# Supplementary figures and images for: Metagenomic and metabolomic analysis showing the adverse risk–benefit trade-off of the ketogenic diet
Source: Lipids Health Dis. 2024 Jun 29;23:207. doi: 10.1186/s12944-024-02198-7 (PMC11218088; doi:10.1186/s12944-024-02198-7)

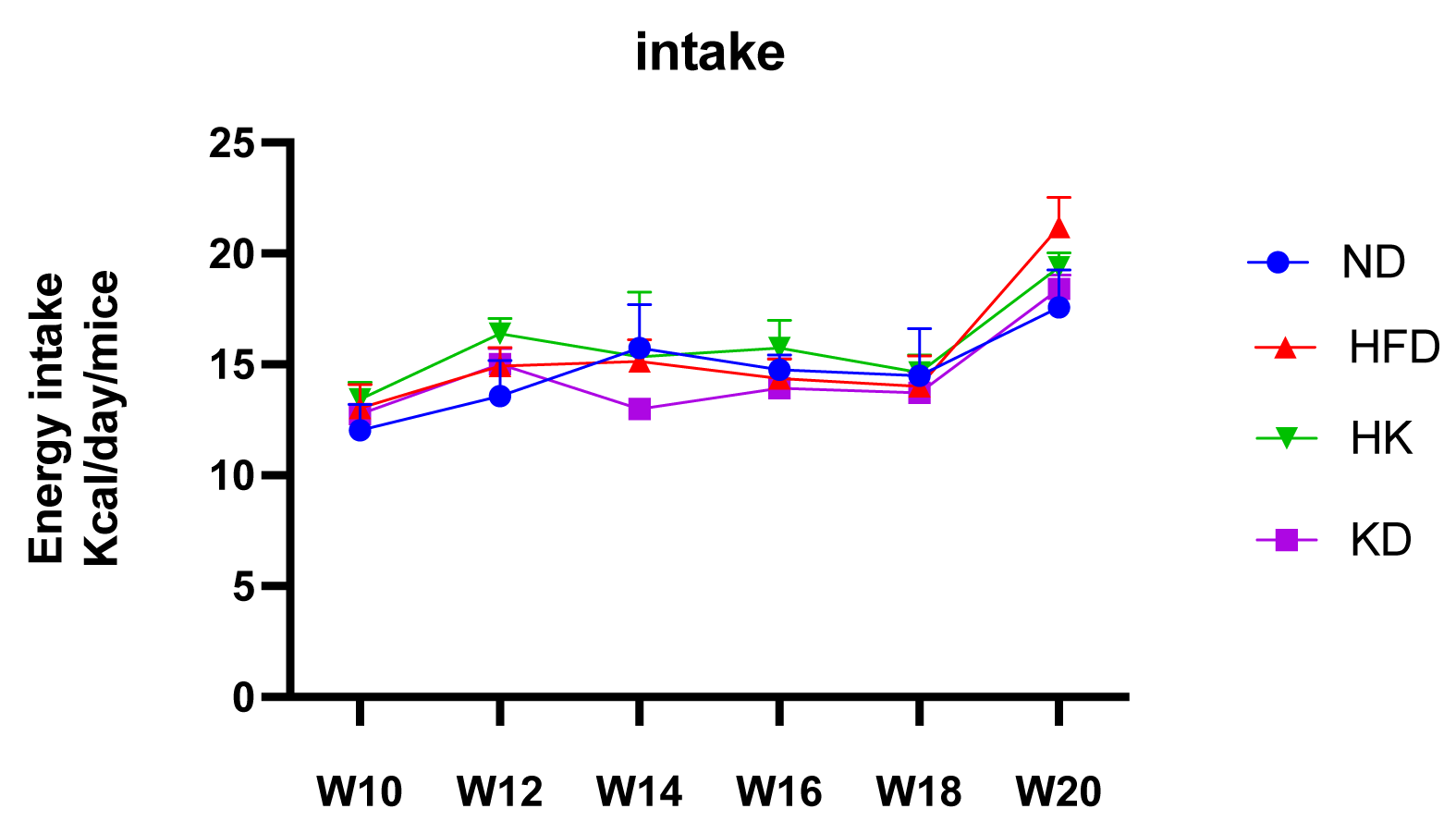

Supplement: Supplementary file 5 — Supplementary Material 5 [file 12944_2024_2198_MOESM5_ESM.png]

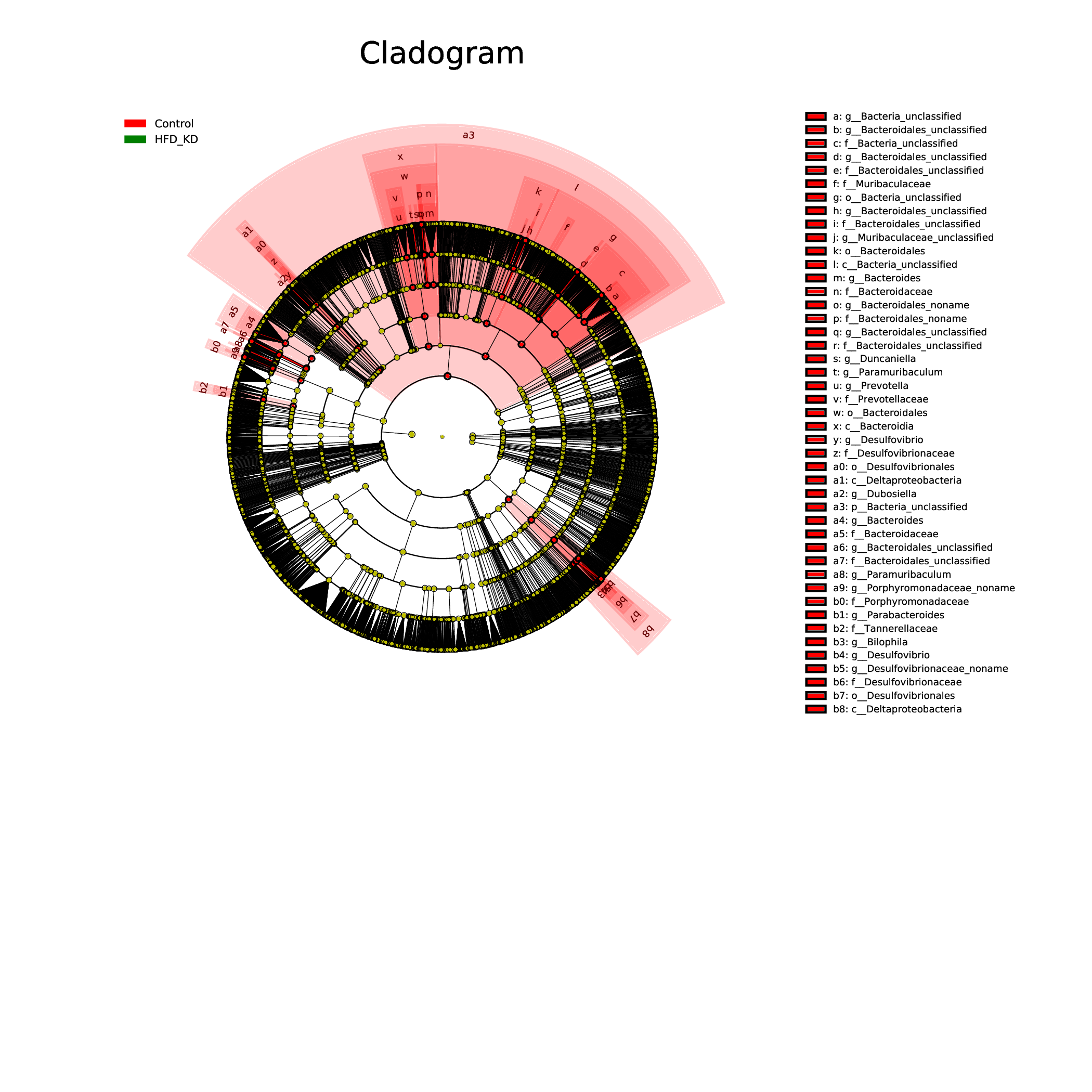

Supplement: Supplementary file 6 — Supplementary Material 6 [file 12944_2024_2198_MOESM6_ESM.png]

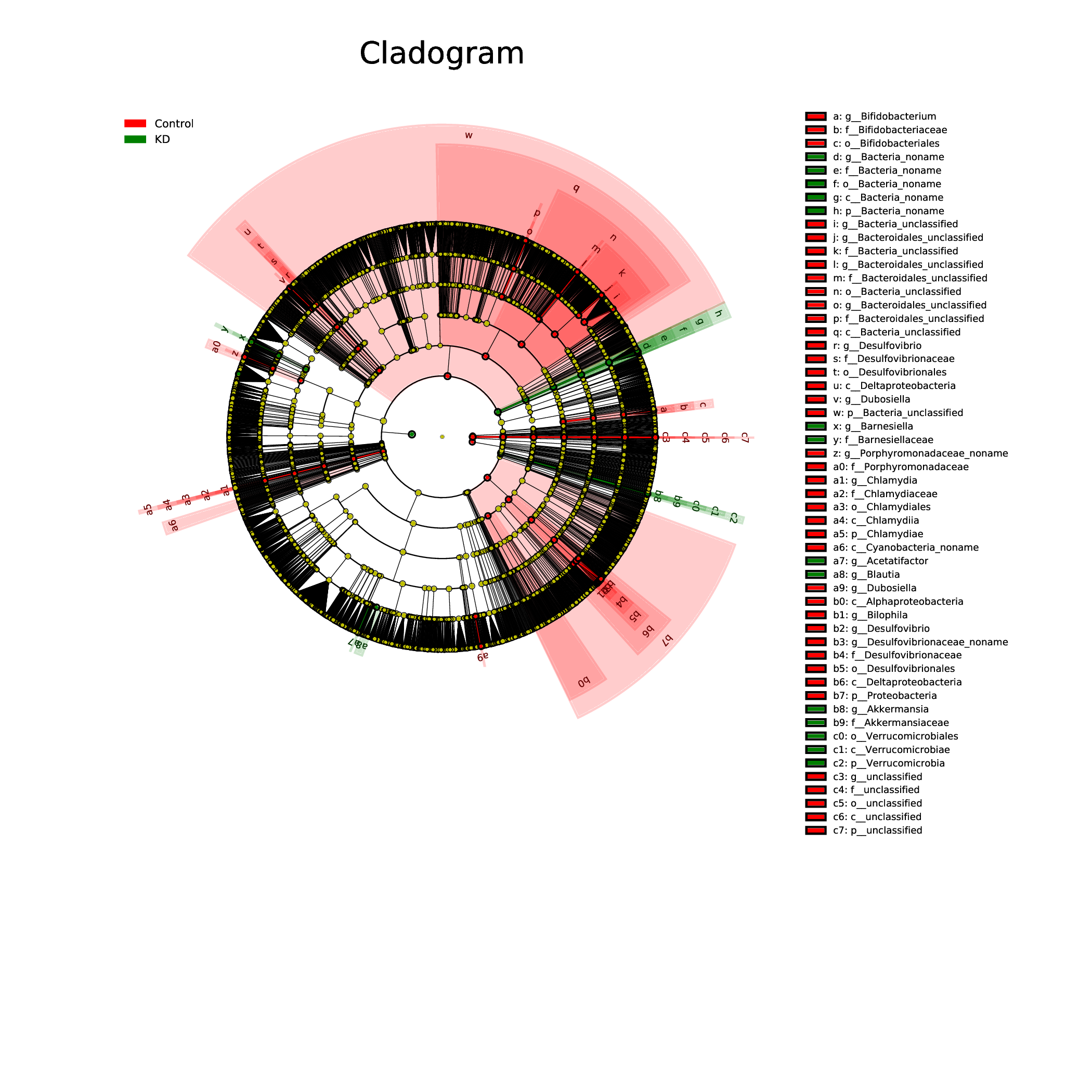

Supplement: Supplementary file 7 — Supplementary Material 7 [file 12944_2024_2198_MOESM7_ESM.png]

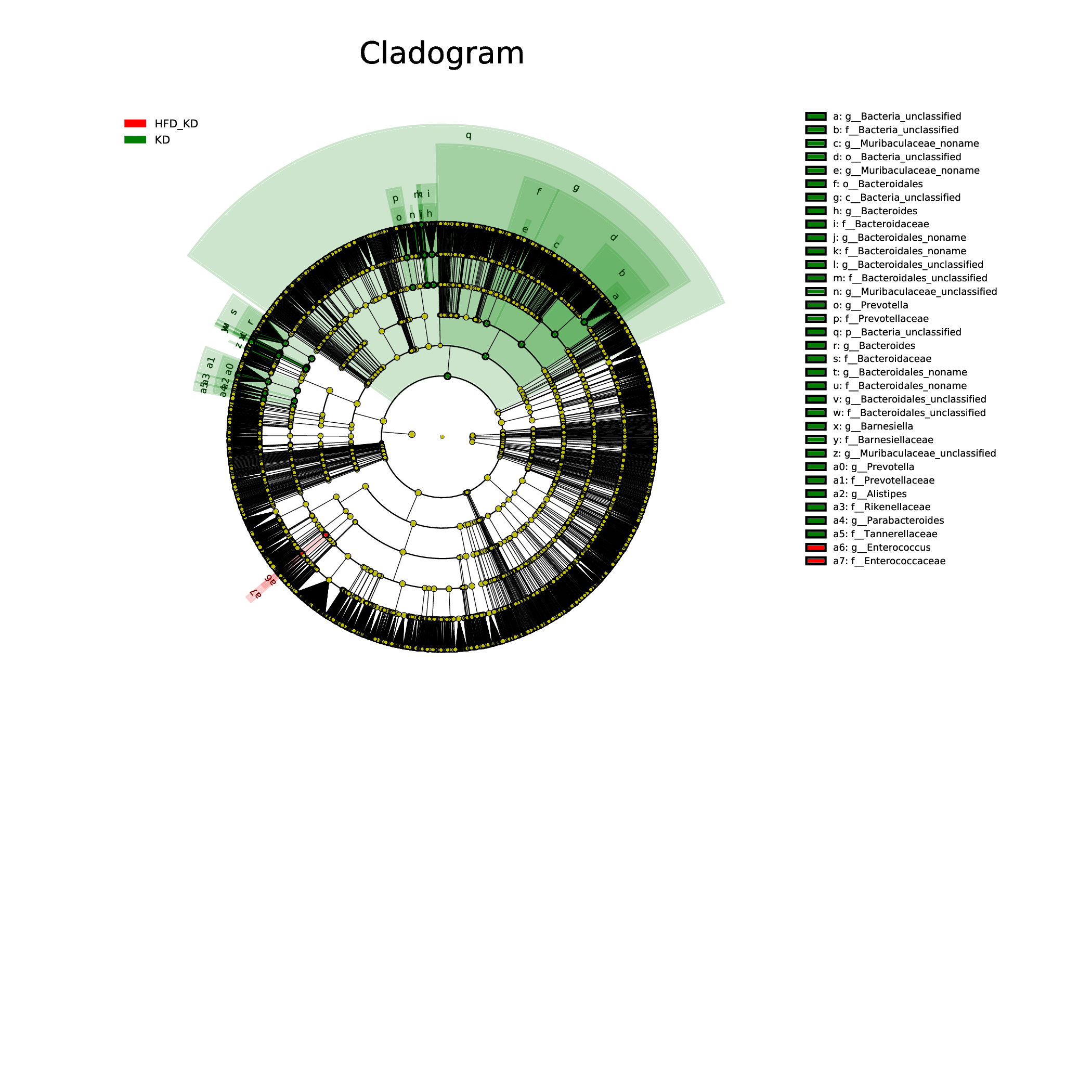

Supplement: Supplementary file 8 — Supplementary Material 8 [file 12944_2024_2198_MOESM8_ESM.png]

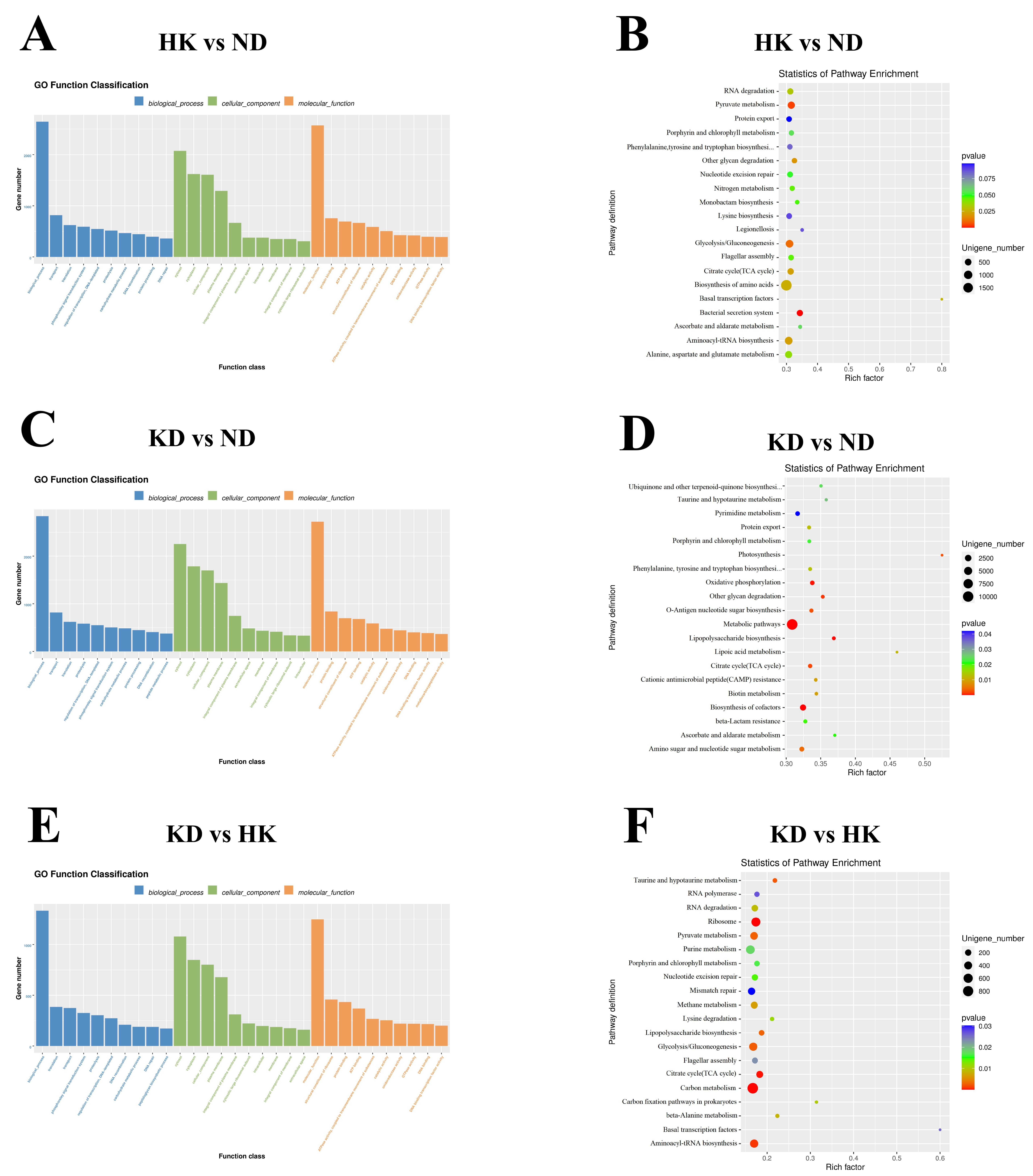

Supplement: Supplementary file 10 — Supplementary Material 10 [file 12944_2024_2198_MOESM10_ESM.tif]

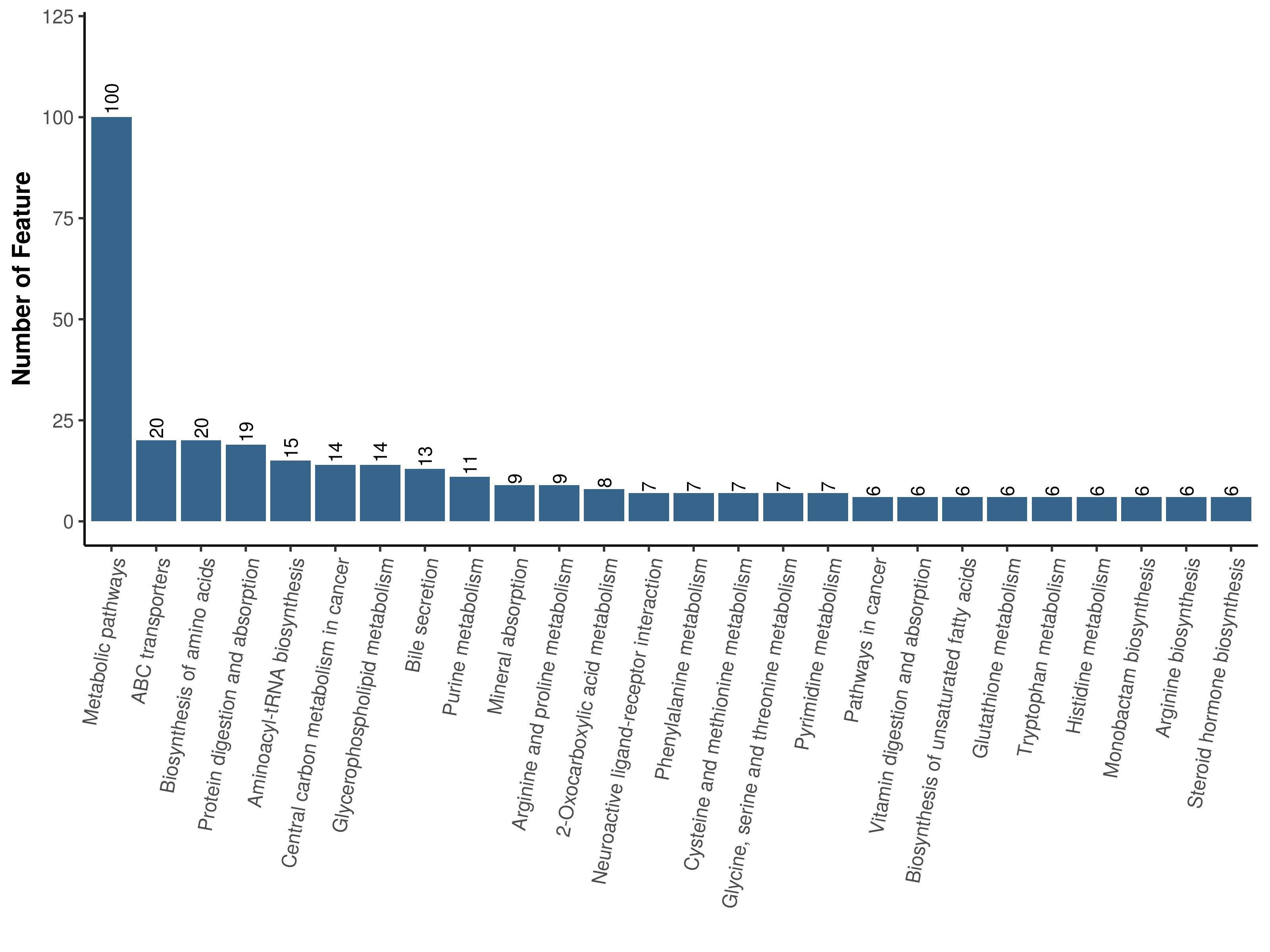

Supplement: Supplementary file 11 — Supplementary Material 11 [file 12944_2024_2198_MOESM11_ESM.png]

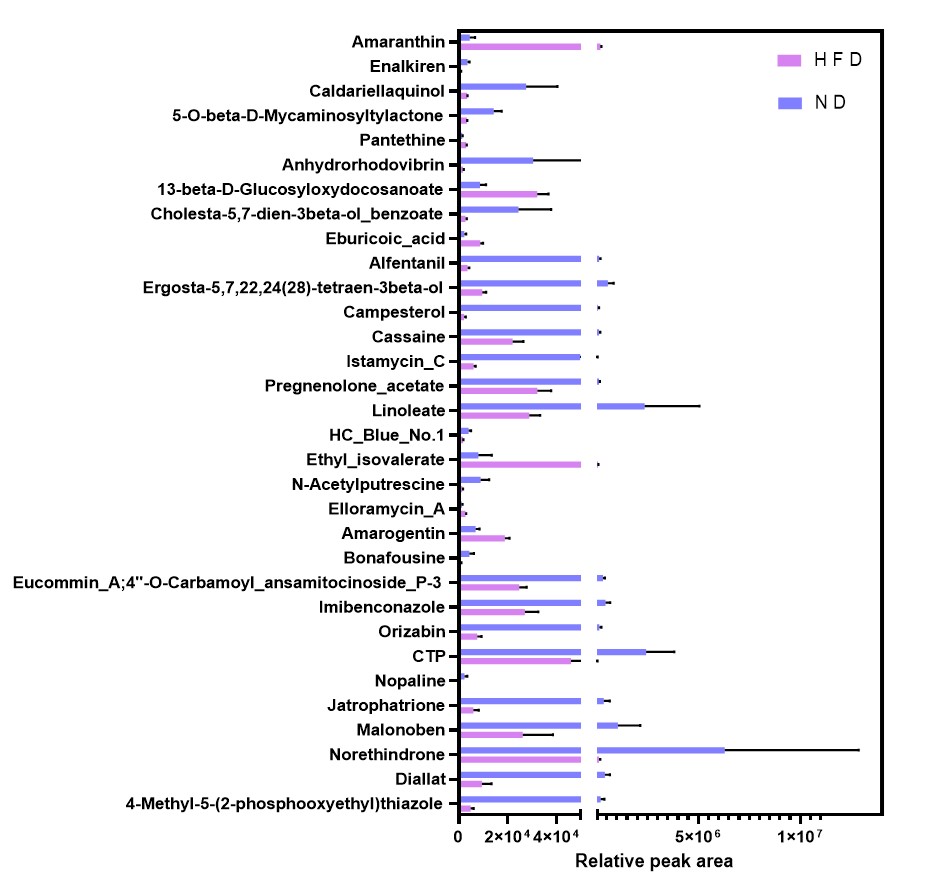

Supplement: Supplementary file 12 — Supplementary Material 12 [file 12944_2024_2198_MOESM12_ESM.jpg]

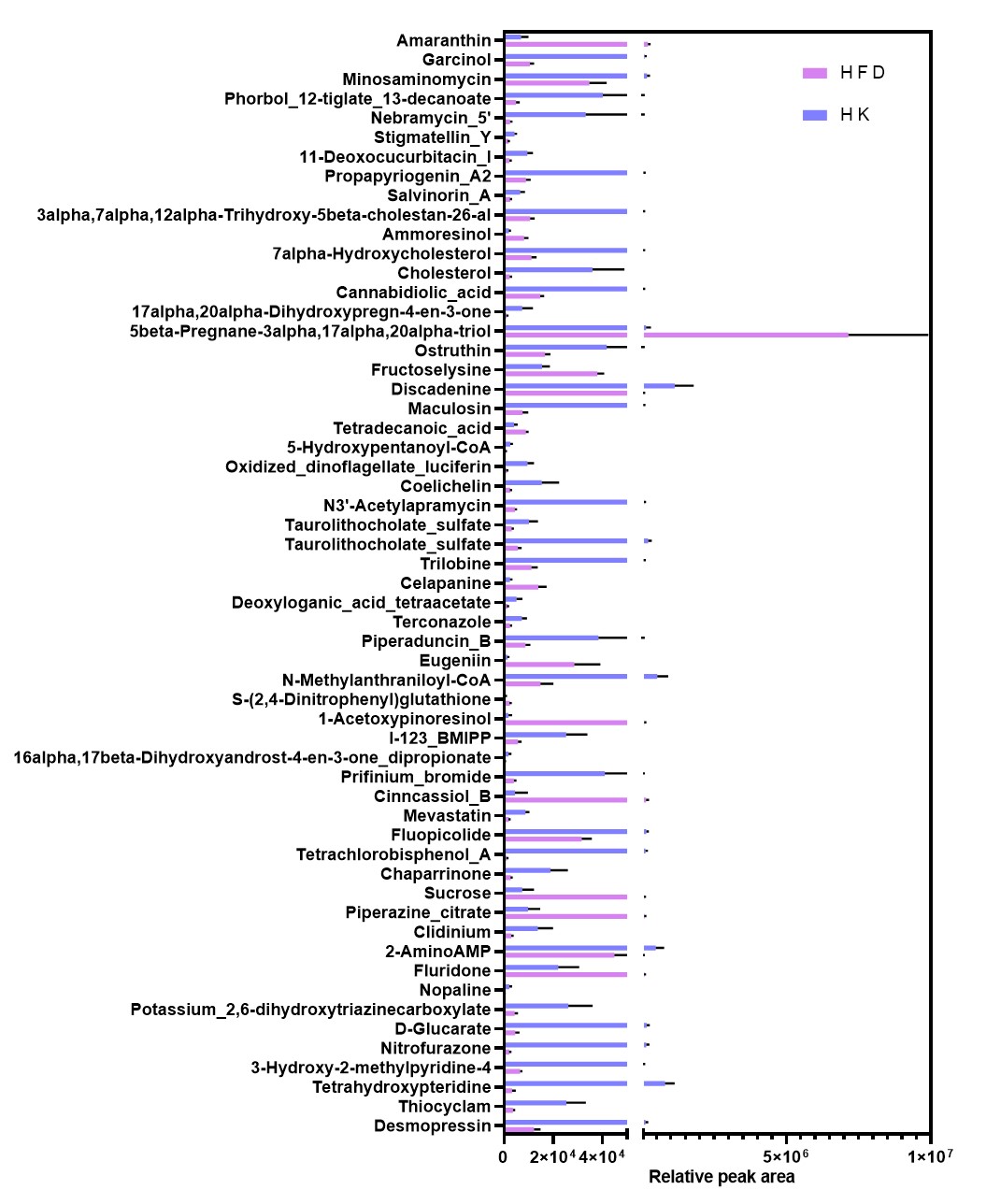

Supplement: Supplementary file 13 — Supplementary Material 13 [file 12944_2024_2198_MOESM13_ESM.jpg]

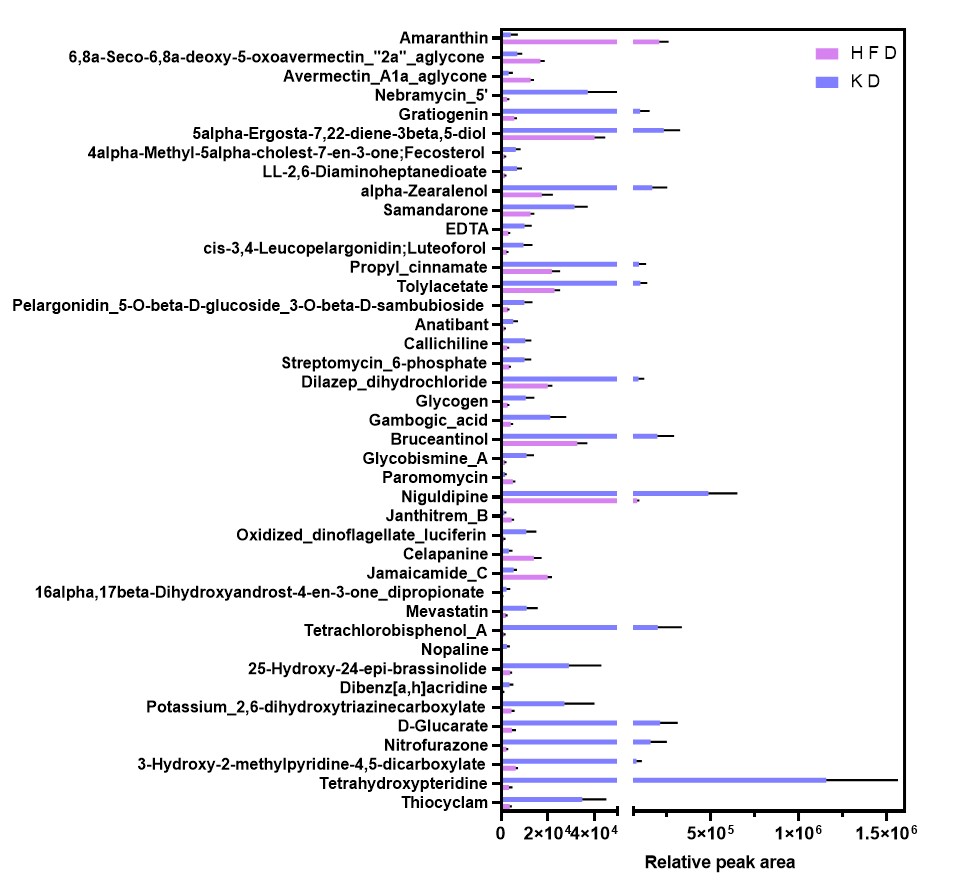

Supplement: Supplementary file 14 — Supplementary Material 14 [file 12944_2024_2198_MOESM14_ESM.jpg]
