# Supplementary material for: Metagenomic and metabolomic analysis showing the adverse risk–benefit trade-off of the ketogenic diet
Source: Lipids Health Dis. 2024 Jun 29;23:207. doi: 10.1186/s12944-024-02198-7 (PMC11218088; doi:10.1186/s12944-024-02198-7)
Supplement: Supplementary file 15 — Supplementary Material 15 [file 12944_2024_2198_MOESM15_ESM.docx]

**Supplementary Figure S1:** Food intake of mice (n=8). This figure illustrates the average daily food intake measured in Kcal for the group of mice over the study period. Data are represented as mean ± SEM.

**Supplementary Figure S2:** LEfSe generated cladogram to identify specific bacterial species in ND and HK group (n=3). This cladogram, generated using Linear discriminant analysis Effect Size (LEfSe), highlights the taxonomic differences in the gut microbiota between the normal diet (ND) and high-fat to ketogenic conversion (HK) groups. The diagram displays the phylogenetic relationships among bacterial taxa, with nodes representing different bacterial species.

**Supplementary Figure S3:** LEfSe generated cladogram to identify specific bacterial species in ND and KD group (n=3). Similar to Supplementary Figure S2, this cladogram illustrates the differences in bacterial species between the normal diet (ND) and ketogenic diet (KD) groups. LEfSe analysis identifies bacterial taxa with significant differences between two groups, represented by colored nodes, showing how the ketogenic diet alters the gut microbiota compared to the normal diet. The phylogenetic tree structure provides insights into the taxonomic levels at which these differences occur.

**Supplementary Figure S4:** LEfSe generated cladogram to identify specific bacterial species in KD and HK group (n=3). LEfSe analysis is used to determine the specific bacterial species between these two diet regimes. The figure shows a phylogenetic tree with nodes color-coded to elucidate the impact of increasing ketogenic dietary content on gut microbiome diversity and composition.

**Supplementary Figure S5**: The heat maps of the differentially expressed genes. A, differentially expressed genes between ND and HFD (n=3); B, differentially expressed genes between HK and HFD (n=3); C, differentially expressed genes between KD and HFD (n=3); D, differentially expressed genes between ND and KD (n=3); E, differentially expressed genes between ND and HK (n=3); F, differentially expressed genes between HK and KD (n=3).

**Supplementary Figure S6**: Enrichment of GO function and KEGG pathway of differentially expressed genes. A, the functions of differentially expressed genes between HK and ND groups (n=3); B, the altered metabolic pathways between HK and ND groups (n=3); C, the functions of differentially expressed genes between KD and ND groups (n=3); D, the altered metabolic pathways between KD and ND groups (n=3). E, the functions of differentially expressed genes between HK and KD groups (n=3); F, the altered metabolic pathways between HK and KD groups (n=3).

**Supplementary Figure S7**: Main metabolic pathways involved in a certain number of metabolites identified by MS2 (n=6).

**Supplementary Figure S8:** Quantitative information of metabolites with significant differences between HFD and ND groups (n=6), data were expressed as mean ± SEM.

**Supplementary Figure S9:** Quantitative information of metabolites with significant differences between HFD and HK groups (n=6), data were expressed as mean ± SEM.

**Supplementary Figure S10:** Quantitative information of metabolites with significant differences between HFD and KD groups (n=6), data were expressed as mean ± SEM.

**Supplementary Table 1**: A more precise visualization of the data shown in Figure 1, including the assessment of body weight, fat mass and fat/weight percentage (n=8).

**Supplementary Table 2**: The analysis result of the carbohydrate-related enzymes of four groups.

**Supplementary Table 3**: Different features identified by MS2 in the metabolomics analysis (n=6).

**Supplementary Table 4**: Metabolites information quantified form LC-MS/MS analysis (n=6).
